# A Whole-Body Mathematical Model of Sepsis Progression and Treatment Designed in the BioGears Physiology Engine

**DOI:** 10.3389/fphys.2019.01321

**Published:** 2019-10-18

**Authors:** Matthew McDaniel, Jonathan M. Keller, Steven White, Austin Baird

**Affiliations:** ^1^Applied Research Associates, Littleton, CO, United States; ^2^Pulmonary and Critical Care Medicine, WISH Simulation Center, University of Washington, Seattle, WA, United States; ^3^Applied Research Associates, Raleigh, NC, United States

**Keywords:** sepsis, sepsis models, sepsis markers, sepsis management, inflammatory models, whole-body modeling, open-source programming tools

## Abstract

Sepsis is a debilitating condition associated with a high mortality rate that greatly strains hospital resources. Though advances have been made in improving sepsis diagnosis and treatment, our understanding of the disease is far from complete. Mathematical modeling of sepsis has the potential to explore underlying biological mechanisms and patient phenotypes that contribute to variability in septic patient outcomes. We developed a comprehensive, whole-body mathematical model of sepsis pathophysiology using the BioGears Engine, a robust open-source virtual human modeling project. We describe the development of a sepsis model and the physiologic response within the BioGears framework. We then define and simulate scenarios that compare sepsis treatment regimens. As such, we demonstrate the utility of this model as a tool to augment sepsis research and as a training platform to educate medical staff.

## Introduction

Sepsis represents an array of dysregulated physiologic responses from the body in response to suspected or confirmed infection. The physiologic changes are often overwhelming, can result in severe tissue damage, and are potentially life-threatening. Large cohort studies indicate sepsis is a leading contributor to hospital mortality (Liu et al., 2014). Patients who acquire sepsis in the hospital have a mortality rate of 25% (Rhee et al., 2017). Sepsis also accounts for 6.2% of the aggregate costs for all hospitalizations, or nearly $23.7 billion annually, making it the most expensive condition treated in the hospital (Torio and Moore, 2016). There is also increasing recognition of sepsis survivors experiencing long-term disability (Iwashyna et al., 2010).

Early identification of septic patients and rapid intervention with appropriate management is imperative. Suspected infection criteria and the Sequential Organ Failure Assessment (SOFA) score were introduced as part of Sepsis-3 (Singer et al., 2016), replacing the previous definitions of sepsis and septic shock last revised in 2001 (Levy et al., 2003). While the Sepsis-3 definitions reflect improved understanding of sepsis pathobiology and improved specificity, no single sepsis definition fully captures the complexity of the syndrome. Numerous potential mediators, host responses, and organ system interactions affect the evolution of sepsis within an individual patient. For similar reasons, there is no universally accepted management protocol for sepsis. Three large randomized controlled trials have not shown protocol-based resuscitation methods improved outcome compared to standard of care (ARISE Investigators and the ANZICS Clinical Trials Group, 2014; ProCESS Investigators, 2014; Mouncey et al., 2015).

Outcomes for septic patients will not improve until we attain a better understanding of the disease and how to treat it. To this end, mathematical modeling can provide significant insight and momentum. While models cannot replace clinical trials, they can drive hypothesis development, narrow the focus of research, and complement medical education. The simplest mathematical models of sepsis typically describe a three- to four-dimensional system comprised of a combination of early and late immune responses, a native anti-inflammatory response, and a measurement of tissue damage (Kumar et al., 2004; Reynolds et al., 2006; Zuev et al., 2006; Jarrett et al., 2015; Caudill and Lynch, 2018). Other, higher-dimensional, models discretize the stages of inflammation into mass-action relationships involving specific pro- and anti-inflammatory mediators that have been implicated in sepsis. These models focus on varying aspects of systemic inflammation such as macrophage recruitment (Smith et al., 2011; Schirm et al., 2016), coagulation (Kumar, 2004), hypotension secondary to nitric oxide (NO) accumulation (Kumar, 2004; Chow et al., 2005; Brady, 2017), endothelial and epithelial tissue barrier characteristics (Reynolds, 2008; Domínguez-Hüttinger et al., 2017), and adaptive immunity (Shi et al., 2015). Most models published in this field consist of ever-growing systems of ordinary or partial differential equations, though some employ stochastic (Song et al., 2012) or machine learning (Mai et al., 2015) techniques.

Though informative, these models by and large do not comprehensively relate the systemic inflammation cascade to observable clinical physiology. For instance, models that describe the relationship between inflammation and blood pressure are generally empirical (Kumar, 2004; Chow et al., 2005; Brady, 2017). We have demonstrated that a physics-based, lumped parameter cardiopulmonary engine can be hybridized with a mathematical model of systemic inflammation to realistically simulate the observable progression of sepsis. The physiology engine in question—BioGears—contains numerous validated systems and feedback models capable of capturing complex and dynamic physical interactions. BioGears also models numerous intervention aspects, such as fluid resuscitation and drug administration, meaning that detailed sepsis treatment scenarios can be investigated for educational, research, and training purposes. Furthermore, BioGears is provided as open-source software, so the methods described herein can be replicated and extended by interested users.

## Materials and Methods

### Overview of BioGears Architecture and Design

**Figure 1:**
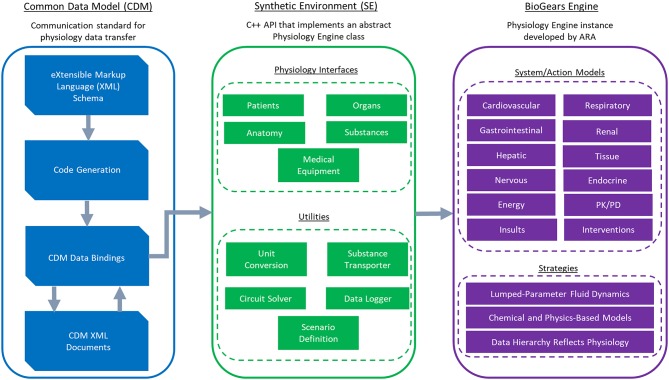
An overview of the BioGears project. The CDM stores BioGears data using an extensible schema definition (XSD) and transfers it in XML format. XSD elements are convereted to data bindings from which the Synthetic Environment (SE) is derived. The SE defines an abstract physiology application programming interface (API). This API contains interfaces of physiological relevance and of convenience utilities. The BioGears Engine is the implementation of the synthetic API being developed by Applied Research Associates. The BioGears Engine contains mechanistic models describing major physiological systems and action models describing the effects of physiological insults (e.g., sepsis and hemorrhage) and interventions (e.g., drugs and fluid infusion).

BioGears—an open-source project developed in C++ by Applied Research Associates—is a virtual physiological human model created with the intention of advancing medical training and research. It consists of three core components: the Common Data Model (CDM), the Synthetic Environment (SE), and the BioGears Engine (Figure 1). The source code, build instructions, bug reporter, and community forum can be found at https://github.com/BioGearsEngine.

#### Common Data Model

The CDM promotes input/output (I/O) transparency by establishing a communication standard for the transfer of physiology data. The current implementation uses an Extensible Schema Definition (XSD) to create a well-defined interface in a common data interchange format. Using XSD allows CDM applications to be stored in the human-readable and self-describing Extensible Markup Language (XML). Additionally, multiple open-source libraries exist for converting XSD to native data bindings that generate the code required to interact with and read the CDM, reducing the amount of interaction required with the schema.

#### Synthetic Environment

The Synthetic Environment is an object-oriented, C++ based Application Programming Interface (API) that implements an abstract Physiology Engine class. This layer of abstraction makes the Physiology Engine extendable to any number of engine instances of varying fidelity. In addition, the SE includes abstract interfaces for patient definition, physiological concepts (such as substances, organs, and systems), numerical solvers, and a unit safe scalar implementation that supports basic dimensional analysis. The SE solvers supported at present pertain specifically to lumped-parameter models (see section BioGears Engine), but the API does not strictly enforce them. Developers can therefore implement physiology engines with different numerical underpinnings and retain compatibility with BioGears-based integrations. As a further convenience, the CDM represents all SE objects, so developers automatically receive full support for physiology state serialization to disk by using the SE as the basis for their engine.

#### BioGears Engine

We refer to our specific Physiology Engine instance as the BioGears Engine. The heart—so to speak—of the BioGears Engine is an electric circuit analog characterizing the fluid dynamics of the cardiopulmonary system. Such circuit representations have a storied history in physiology, beginning with the *windkessel* model developed by Otto Frank to describe cardiac pumping (Frank, 1899). Frank's original *windkessel* contained two elements: a resistor representing vessel resistance to flow and a capacitor representing vessel compliance. Subsequent work led to the addition of a second resistor (the three-element *windkessel*) and an inductor (four-element *windkessel*) to capture aortic impedance (Westerhof et al., 2008).

While *windkessel* models originally represented system-wide flow characteristics, BioGears uses a series of connected *windkessels* to model blood flow in each organ and in major vessels. These organ-level *windkessels* for the most part contain three elements (two resistors and a capacitor), though some organs like the kidneys have expanded circuit levels to increase fidelity. Pumping of the heart occurs by adjusting the compliance of the cardiac *windkessel* according to validated pressure-volume data. A similarly constructed, though smaller, circuit comprises the BioGears respiratory system, which also contains diffusion models defining gas exchange. Collectively, these circuits constitute a lumped parameter, or “zero-dimensional,” system. That is, because no spatial component exists, the pressure, volumes, and flows calculated on each circuit represent values averaged (lumped) by organ. Such an approach is appropriate for a model of this size considering the computational cost incurred by increasing fidelity. Higher dimensional models require numerical solution of some form of the Navier-Stokes equations (either the full system or a simplification assuming, for instance, radial symmetry or low Reynolds number) (Batchelor, 1970; Olufsen and Nadim, 2004). Given that BioGears aims to support simulation at speeds faster than real time, incorporating these models is not within the scope of this effort. Future research considering a multi-scale cardiovascular system would be one possible bridge between modeling paradigms.

The BioGears Engine organizes lumped data hierarchically into compartments. Top-level compartments generally represent organs or systems, with sub-compartments representing entities such as the vascular, tissue, extracellular, and intracellular spaces. All compartments collectively associated with a circuit constitute a graph. The engine maintains compartment overlays and circuit-to-graph mapping by implementing a Compartment Manager class defined by the SE. Each simulation cycle, the BioGears Engine solves all circuit states using an SE numerical solver. The Compartment Manager then pulls information from the circuit to determine substance fluxes across its associated graph. For instance, the engine calculates oxygen transfer between the heart and aorta compartments on the cardiovascular graph by querying the flow across the heart to aorta path on the cardiovascular circuit. If provided, user-defined patient parameters, such as heart rate (HR), systolic blood pressure (SBP), diastolic blood pressure (DBP), and respiration rate (RR), determine the baseline state of all circuits and graphs; otherwise the engine defaults to standard values.

Numerous chemical and physics-based models have been built upon this backbone to produce a fast and accurate whole-body physiology model. Examples of such models include: a rudimentary nervous system with baroreceptor and chemoreceptor feedback that modifies cardiovascular and respiratory activity; an active transport model that maintains ionic gradients across intracellular and extracellular compartments; a physiologically-based pharmacokinetic/pharmacodynamic (PBPK/PD) model that tracks the concentration-effect profile of numerous drugs; a gastrointestinal model that determines rates of nutrient digestion and oral drug absorption; a renal feedback model that regulates urine production and substance filtration and reabsorption; and a metabolic consumption and production method that determines the energy demands of each organ. Furthermore, numerous actions, insults, and conditions can be applied to the system, ranging from acute hemorrhage to diabetes. Detailed documentation of these models and actions can be found at https://biogearsengine.com/. Model development is ongoing—as this paper demonstrates—and the documentation will be updated appropriately.

### BioGears Sepsis Model

#### Acute Inflammatory Response (AIR) Model

We based our initial model of inflammation in BioGears on the diverse shock model of Chow et al. (2005). Though optimized using murine data, this model considers a wide range of pro- and anti-inflammatory mediators implicated in human models of inflammation, such as tumor necrosis factor alpha (TNF) and interleukins 6 and 10 (IL-6, IL-10) (Zhang and An, 2007). Consideration of these factors in conjunction with activation of macrophages and neutrophils increases the variability in virtual patient outcomes supported by the model. Furthermore, the diverse shock model explores the role of nitric oxide in blood pressure homeostasis, a pathway of great interest in septic shock research (Vincent et al., 2000). This model also lends itself well to the BioGears methodology due to its ability to simulate inflammation of varying origins. Indeed, we have already used this framework to implement a model of burn-induced systemic inflammation in BioGears.

Chow et al. developed their model assuming an exogenously administered endotoxin to be the driving force of inflammation. As such, the diverse shock model does not consider an actively growing bacteria population. We therefore introduced the model of bacterial colonization and invasion derived by Domínguez-Hüttinger et al. (2017) to the BioGears inflammation model. This invasion model assumes a *Streptococcus pneumoniae* inoculum colonizes in the lungs and diffuses across the apical epithelium into the bloodstream. The model tracks the integrity of the epithelial barrier, which transiently decreases to allow neutrophil recruitment to the lungs at the risk of enhanced bacterial migration to the blood. Deterioration of the epithelium and neutrophil transmigration is managed by a switch that represents the activity of toll-like receptors (TLR). We use the bacteria count in the blood tracked by this model as the input to the diverse shock model. Furthermore, we assume that the immune mediators associated with the invasion and diverse shock models represent local tissue counts and systemic blood counts, respectively. We also subject the blood-born bacteria to phagocytosis by blood neutrophils, similarly to the approach taken in Reynolds (2008). Finally, we drop the assumption of strep colonization of the lungs and assume rather a generic bacteria infiltrating a tissue space.

We produce the full model here and note additional minor modifications made to specific equations below. See Table 1 for descriptions of state variables and Table 2 for parameter values.

(1) $$\begin{array}{l} \frac{dP_{T}}{dt}=\left(\frac{S_{P}}{k_{P_{T}}}\right)\;\cdot P_{T}\;\cdot \;\left(1-P_{T}\right)-\frac{\theta_{P}\;\cdot \;P_{T}}{1\;+\;k_{P_{T}B}\;\cdot \;P_{T}}\\ \;-\;k_{P_{T}M_{T}}\;\cdot \;M_{T}\;\cdot \;P_{T}-k_{P_{T}N_{T}}\;\cdot \;N_{T}\;\cdot \;P_{T} \end{array}$$

(2) $$\begin{array}{lll} \frac{dM_{T}}{dt} & = & \frac{S_{M_{T}}\;\cdot \;N_{T}\;\cdot \;M_{v}}{1\;+\;k_{M_{T}B}\;\cdot \;B}-k_{M_{T}}\cdot M_{T} \end{array}$$

(3) $$\begin{array}{lll} \frac{dN_{T}}{dt} & = & \frac{S_{N_{T}}\;\cdot \;R\;\cdot \;N_{v}}{\left(1\;+\;k_{N_{T}B}\;\cdot \;B\right)\;\cdot \;\left(1\;+\;k_{N_{T}M_{T}}\;\cdot \;M_{T}\right)}-k_{N_{T}}\cdot N_{T} \end{array}$$

(4) $$\begin{array}{lll} \frac{dB_{T}}{dt} & = & \frac{S_{B}}{1\;+\;k_{BP_{T}}}\cdot B_{T}\cdot \left(1-B_{T}\right)-k_{BR}\cdot R\cdot B_{T}-\;k_{BN_{T}}\cdot N_{T}\cdot B_{T} \end{array}$$

(5) $$\begin{array}{l} \frac{dP_{B}}{dt}=S_{P}\cdot P_{B}\;+\;\frac{\theta_{P}\;\cdot \;P_{T}}{1\;+\;k_{P_{T}B}\;\cdot \;P_{T}}-\frac{k_{PS}\;\cdot \;P_{B}}{x_{PS}\;+\;P_{B}}\\ \;-\;k_{P_{B}N_{A}}\cdot N_{A}\cdot H_{U2}\left(P_{B},\;x_{PN},2\right) \end{array}$$

(6) $$\begin{array}{l} \frac{dM_{R}}{dt}=-[\left(k_{MP}\cdot H_{U2}\left(P_{B},x_{MP},2\right)+k_{MD}\cdot H_{U2}\left(1-TI,x_{MD},4\right)\right)\\ \;\cdot \left(H_{U2}\left(TNF,x_{MTNF},2\right)+k_{M6}\cdot H_{U2}\left(IL6,x_{M6},2\right)\right)]\\ \;\cdot H_{D}(IL10,x_{M10},2)\cdot M_{R}-k_{MR}\cdot (M_{R}-S_{M}) \end{array}$$

(7) $$\begin{array}{l} \frac{dM_{A}}{dt}=[\left(k_{MP}\cdot H_{U2}\left(P_{B},x_{MP},2\right)+k_{MD}\cdot H_{U2}\left(1-TI,x_{MD},4\right)\right)\\ \;\cdot \;\left(H_{U2}\left(TNF,x_{TNF},2\right)+k_{M6}\cdot H_{U2}\left(IL6,x_{M6},2\right)\right)]\\ \;\cdot \;H_{D}\left(IL10,x_{M10},2\right)\;\cdot M_{R}-k_{M_{A}}\cdot M_{A} \end{array}$$

(8) $$\begin{array}{l} \frac{dN_{R}}{dt}=-(k_{NP}\cdot H_{U2}\left(P_{B},x_{NP},1\right)+k_{ND}\cdot H_{U1}\left(1-TI,x_{ND},2\right)\\ \;+k_{NTNF}\cdot H_{U1}\left(TNF,x_{NTNF},1\right)+k_{N6}\cdot H_{U1}\left(IL6,x_{N6},2\right))\\ \;\cdot \;H_{D}\left(IL10,x_{N10},2\right)\cdot N_{R}-k_{N_{R}}\cdot (N_{R}-S_{N}) \end{array}$$

(9) $$\begin{array}{l} \frac{dN_{A}}{dt}=(k_{NP}\cdot H_{U2}\left(P_{B},x_{NP},1\right)+k_{ND}\cdot H_{U1}\left(1-TI,x_{ND},2\right)\\ \;+k_{NTNF}\cdot H_{U1}\left(TNF,x_{NTNF},1\right)+k_{N6}\cdot H_{U1}\left(IL6,x_{N6},2\right))\\ \;\cdot H_{D}\left(IL10,x_{N10},2\right)\cdot N_{R}-k_{N_{A}}\cdot N_{A} \end{array}$$

(10) $$\begin{array}{l} \frac{diNOSd}{dt}=(k_{INOSN}\cdot N_{A}+k_{INOSM}\cdot M_{A}+k_{INOSEC}\cdot \\ \;\left(H_{U1}(TNF,x_{INOSTNF},2)+k_{INOS6}\cdot H_{U1}(IL6,x_{INOS6},2)\right))\\ \;\cdot \;H_{D}\left(IL10,x_{INOS10},2\right)\cdot \;H_{D}\left(NO,x_{INOSNO},4\right)\\ \;-k_{INOSd}\cdot iNOSd \end{array}$$

(11) $$\begin{array}{lll} \frac{diNOS}{dt} & = & k_{INOS}\cdot \left(iNOSd-iNOS\right) \end{array}$$

(12) $$\begin{array}{l} \frac{deNOS}{dt}=k_{ENOSEC}\cdot H_{D}\left(TNF,x_{ENOSTNF},1\right)\;\cdot H_{D}\left(P_{B},x_{ENOSP},1\right)\\ \;-k_{ENOS}\cdot \;eNOS \end{array}$$

(13) $$\begin{array}{lll} \frac{dNO3}{dt} & = & k_{NO3}\cdot \left(NO-NO3\right) \end{array}$$

(14) $$\begin{array}{l} \frac{dTNF}{dt}=\left(k_{TNFN}\cdot N_{A}+k_{TNFM}\cdot M_{A}\right)\;\cdot \;H_{D}\left(IL10,x_{TNF10},2\right)\\ \;\cdot \;H_{D}\left(IL6,x_{TNF6},3\right)-k_{TNF}\cdot \;TNF \end{array}$$

(15) $$\begin{array}{l} \frac{dIl6}{dt}=\left(k_{6N}\cdot N_{A}+M_{A}\right)\;\cdot \;(k_{6M}+k_{6TNF}\cdot H_{U2}\left(TNF,x_{6TNF},2\right)\\ \;+\;k_{6NO}\cdot \;H_{U2}\left(NO,x_{6NO},2\right))\;\cdot \;H_{D}\left(IL10,x_{610},2\right)\\ \;\cdot H_{D}\left(IL6,x_{66},1\right)-k_{6}\cdot (IL_{6}-S_{6}) \end{array}$$

(16) $$\begin{array}{l} \frac{dIl10}{dt}=\left(k_{10N}\cdot N_{A}+M_{A}\right)\;\cdot \;(k_{10MA}+k_{10TNF}\cdot H_{U2}\left(TNF,x_{10TNF},4\right)\\ \;+\;k_{106}\cdot \;H_{U2}\left(IL6,x_{106},4\right))\;\cdot \;(\left(1-k_{10R}\right)\\ \;\cdot H_{D}\left(IL12,x_{1012},4\right)+k_{10R})-\;k_{10}\cdot \;(IL_{10}-S_{10}) \end{array}$$

(17) $$\begin{array}{lll} \frac{dIL12}{dt} & = & k_{12M}\cdot M_{A}\cdot H_{D}\left(IL10,x_{1210},2\right)-k_{12}\cdot IL12 \end{array}$$

(18) $$\begin{array}{l} \frac{dTI}{dt}=k_{D}\cdot \;\left(1-TI\right)\;\cdot \;\left(TI-TI_{min}\right)\\ \;-\;\left(TI-TI_{min}\right)\;\cdot \;k_{D6}\cdot \;H_{U2}(IL6,x_{D6},6)\\ \;\cdot \;\frac{1}{x_{DNO}^{2}+NO^{2}} \end{array}$$

(19) $$\begin{array}{lll} NO & = & iNOS\cdot \left(1+k_{NOMN}\cdot \left(M_{A}+N_{A}\right)\right)+eNOS \end{array}$$

(20) $$\begin{array}{lll} H_{U1}\left(x,n,h\right) & = & \frac{x^{h}}{1\;+\;{\left(x/n\right)}^{h}} \end{array}$$

(21) $$\begin{array}{lll} H_{U2}\left(x,n,h\right) & = & \frac{x^{h}}{x^{h}\;+\;n^{h}} \end{array}$$

(22) $$\begin{array}{lll} H_{D}\left(x,n,h\right) & = & \frac{1}{1+{\left(x/n\right)}^{h}} \end{array}$$

(23) $$R(t)=\{\begin{array}{c} 1\;\;\;\;\;\;\;\;\;\;\;\;P_{T}>P^{+}\;or\;\left(P^{-}<P_{T}<\;P^{+}\;and\;R\left(t-1\right)=1\right)\\ 0\;\;\;\;\;\;\;\;\;\;\;\;P_{T}<P^{-}\;or\;\left(P^{-}<P_{T}<\;P^{+}\;and\;R\left(t-1\right)=0\right) \end{array}$$

The equation describing blood-born bacterial dynamics (Equation 5) essentially bridges the bacterial invasion model (Equations 1–4, 23) and the diverse shock model (Equations 6–22). We made no further modifications to the invasion model, but Equation (23) merits some discussion. This equation describes the state of the TLR switch that regulates the epithelial barrier integrity of the tissue that the bacteria has infiltrated. In the “on” state, barrier integrity is more easily reduced, which promotes neutrophil migration to the site of infection (Equation 3). Barrier reduction, however, also allows for greater bacterial flux to the bloodstream (Equation 1). The “off” state of the TLR switch supports epithelial healing and limits neutrophil and bacteria movement. Equation (23) states that at high bacteria concentrations (above a predetermined limit P^+^), the switch is always in the “on” mode. Likewise, the switch remains off when the tissue bacteria levels fall below a minimum threshold (P^−^). At moderate bacteria levels (P^−^ < P_T_ < P^+^), the switch remains in its previous state.

**Table 1:** State variables in the Acute Inflammatory Response model based on the work of Chow et al. (2005) and Domínguez-Hüttinger et al. (2017).

**Symbol**	**Description**	**Symbol**	**Description**
P_T_	Bacteria at the site of infection. This value is initialized to begin an infection scenario	iNOS	Inducible nitric oxide synthase, produces nitric oxide
M_T_	Local macrophages at the site of infection	eNOS	Constitutive eNOS, contributes to normal background levels of nitric oxide
N_T_	Local neutrophils at the site of infection	NO_3_	Nitrate, product of nitric oxide
B	The integrity of the local tissue at the site of infection. This barrier must be weakened for neutrophils to migrate to the infection site, but doing so facilitates bacteria translocation to blood	TNF	Tumor necrosis factor, early pro-inflammatory mediator upregulated by M_A_ and N_A_
P_B_	Bacteria that has diffused to the blood from infection site	IL6	Interleukin-6, later pro-inflammatory mediator which also downregulates TNF
M_R_	Resting blood macrophage population, converted to active form by P and other pro-inflammatory mediators	IL10	Interleukin-10, primary anti-inflammatory mediator
M_A_	Activated blood macrophage population	IL12	Interleukin-12, moderates IL10 production
N_R_	Resting blood neutrophil population, converted to active form by P and other pro-inflammatory mediators	TI	Tissue integrity ranging from 1.0 (healthy) to 0.0 (irreversible damage). Depleted by interleukin-6 and pathogen, moderated by NO
N_A_	Active blood neutrophil population	NO	Nitric oxide, exerts hypotensive activity
iNOSd	Precursor to inducible nitric oxide synthase, activated by TNF	S	Non-specific background immune response (implicit in model)

**Table 2:** Parameters for the AIR model based on the work of Chow et al. and Dominguez-Huttinger et al.

**Parameter**	**Value**	**Parameter**	**Value**	**Parameter**	**Value**	**Parameter**	**Value**	**Parameter**	**Value**
*S_*P*_*	0.60^∧^	$P_{T}^{--}$	1e3^∧^	*k_*NTNF*_*	0.2	*x_*ENOSP*_*	1.015	*k_10*MA*_*	0.1
*k*_*P*_*T*__	3.7e4^∧^	*k*_*P*_*B*_*N*_*A*__	5.8^*^	*x_*NTNF*_*	2.0	*k_*ENOS*_*	4.0	*k_10*TNF*_*	1.485
θ_*P*_	1.35e-4^∧^	*x_*PN*_*	0.5^*^	*k_*N*6_*	1.5	*k_*NO*3_*	0.46	*x_10*TNF*_*	0.05
*k*_*P*_*T*_*B*_	3.1^∧^	*k_*PS*_*	6.9e3^∧^	*x_*N*6_*	1.0	*k_*NOMA*_*	2.0	*k_106_*	5.1e-2
*k*_*P*_*T*_*M*_*T*__	6.3e-3^∧^	*x_*PS*_*	1.3e4^∧^	*x_*N*10_*	0.2	*k_*TNFN*_*	2.97	*x_106_*	8.0e-2
*k*_*P*_*T*_*N*_*T*__	6.1e-4^∧^	*k_*MP*_*	1.01	*k*_*N*_*R*__	0.05	*k_*TNFM*_*	0.1	*k_10*R*_*	0.1
*S*_*M*_*T*__	2.6e-2^∧^	*x_*MP*_*	37.5^*^	*S_*N*_*	1.0	*x_*TNF*10_*	7.9e-2	*x_1012_*	1.0e-2^*^
*M*_*v*_	0.3^∧^	*k_*MD*_*	5.0e-2^*^	*k*_*N*_*A*__	0.5	*x_*TNF*6_*	5.9e-2	*k_10_*	0.35
*k*_*M*_*T*__	6.43e-5^∧^	*x_*MD*_*	0.75^*^	*k_*INOSN*_*	1.5	*k_*TNF*_*	1.4	*S_10_*	1.0e-2
*k*_*M*_*T*_*B*_	36.0^∧^	*x_*MTNF*_*	0.4	*k_*INOSM*_*	0.1	*k_6*N*_*	0.2	*k_12*M*_*	0.303
*S*_*N*_*T*__	7.0e-7^∧^	*k_*M*6_*	0.1	*k_*INOSEC*_*	0.1	*k_6*M*_*	3.03	*x_1210_*	0.2525
*N*_*v*_	1e8^∧^	*x_*M*6_*	1.0	*x_*INOSTNF*_*	0.05	*k_6*TNF*_*	1.0	*k_12_*	5.0e-2
*k*_*N*_*T*_*B*_	36.0^∧^	*x_*M*10_*	0.297	*k_*INOSd*_*	0.05	*x_6*TNF*_*	0.1	*k_*D*_*	0.15^*^
*k*_*N*_*T*_*M*_*T*__	0.16^∧^	*k*_*M*_*R*__	0.05	*k_*INOS*6_*	2.0	*k_6*NO*_*	2.97	*k_*D*6_*	0.125^*^
*k*_*N*_*T*__	6.1e-2^∧^	*S_*M*_*	1.0	*x_*INOS*6_*	0.1	*x_6*NO*_*	0.4	*x_*D*6_*	0.85^*^
*S*_*B*_	4.6e-2^∧^	*k*_*M*_*A*__	0.2	*x_*INOS*10_*	0.1	*x_610_*	0.1782	*x_*DNO*_*	0.5^*^
*k*_*B*_*P*__*T*__	26.0^∧^	*k_*NP*_*	33.75	*x_*INOSNO*_*	0.3	*x_66_*	0.5^*^		
*k*_*BR*_	0.14^∧^	*x_*NP*_*	56.25	*k_*INOS*_*	0.101	*k_6_*	0.7		
*k*_*B*_*N*__*T*__	4.0e-8^∧^	*k_*ND*_*	0.05	*k_*ENOSEC*_*	0.05	*S_6_*	1.0e-3		
$P_{T}^{+}$	2.0e6^*^	*x_*ND*_*	0.4	*x_*ENOSTNF*_*	0.4	*k_10*N*_*	0.1		

Parameters marked with

∧ are obtained from Dominguez-Huttinger. Parameters marked with

* *are tuned for BioGears response. All others are as reported in Chow. Conventions S_A_, source of A; k_A_, decay of A; k_AB_, effect of B on A; x_AB_, amount of B that induces half-max effect on A; A_v_, resting pool of A; θ_A_, diffusion rate of A; A^+/−^, A upper/lower threshold. Time scale, 1/hr*.

We modified Equations (6–23) by dropping terms relating to blood pressure, trauma, hemorrhage, and autonomic effects. BioGears already accounts for blood pressure and catecholamine activity (in the form of epinephrine) mechanistically and we did not deem the other effects critical in a model of sepsis. The expression for tissue damage (Equation 18) has been rearranged and scaled after the rfashion of Reynolds (2008) so that tissue damage is bounded between 1 (health) and 0 (irreversible damage). Due to the inversion of boundaries, we have replaced the term Damage (D) used by Chow with Tissue Integrity (TI). This mapping provides a simpler implementation for downstream BioGears functions that accept tissue integrity as an input. Finally, we have included an additional term in Equation 11 to account for IL-6 self-inhibition. We found that modeling this behavior guaranteed the existence of a stable fixed point in the range of pathogen growth rates that we investigated. We henceforward refer to the system in Equations (1–23) as the Acute Inflammatory Response (AIR) model.

We did not perform a formal bifurcation analysis of the AIR model. However, we varied the pathogen growth rate (S_P_) and initial tissue pathogen load (P_T0_) to identify regions in which the model demonstrates bi-stability between “healthy” (bacteria in blood eliminated) and “septic” (bacteria in blood not eliminated) outcomes. We determined that for bacteria growth rates in the range 0.55 < *S*_*P*_ < 0.70 there exists a critical initial tissue bacteria load $P_{T0}^{*}$ such that:

(24) $${\text{lim}}_{t\to \infty }P_{B}\left(t\right)=\{\begin{array}{c} 0\;\;\;\;\;\;\;\;\;\;\;\;\;\;P_{T0}\leq P_{T0}^{*}\\ P_{Bf}\;\;\;\;\;\;\;\;\;\;P_{T0}>P_{T0}^{*}\; \end{array}$$

In Equation 24, *P*_*Bf*_ is some final non-zero blood bacteria count indicative of sepsis. The value of $P_{0}^{*}$ depends on the choice of *S*_*P*_. When, for instance *S*_*P*_ = 0.6, (its nominal value in our model), $P_{T0}^{*}$ is ~ 6.95 · 10^6^ CFU/mL.

#### Endothelial Dysfunction and Hypovolemia

Under normal physiological conditions, the body maintains a slight fluid flux directed from the vasculature to the interstitium. The filtered fluid returns to the cardiovascular system via the lymphatic system, thereby maintaining fluid balance. Inflammation disrupts this balance by stimulating production of compounds that modify the glycocalyx—a protein network on the luminal side of the endothelium—by widening its gaps (Boron and Boulpaep, 2017). This action promotes adhesion of white blood cells to the endothelial wall and hastens their transport into the afflicted tissue (Mulivor and Lipowsky, 2004). However, unchecked pro-inflammatory activity—such as that observed in sepsis—degrades the glycocalyx to the point that it becomes freely permeable (Chelazzi et al., 2015). As a result, large plasma proteins such as albumin leak into the interstitium, disrupting the colloid osmotic pressure (COP) gradient that normally favors fluid retention in the vasculature. Hydrostatic pressure forces become dominant and fluid leaks into the extravascular space, leading to relative hypovolemia.

**Figure 2:**
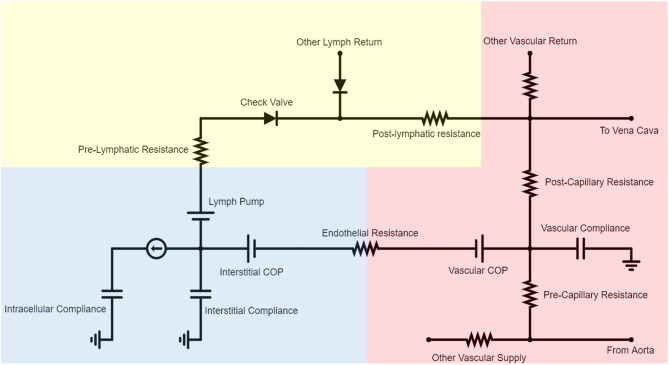
A representative subset of the BioGears Cardiovascular Circuit demonstrating the interaction between each vascular-tissue compartment pair. Most blood flow is restricted to the vascular compartment (red), circulating from the aorta to each organ and returning to the heart via the vena cava. Each organ has a vascular compliance representing its ability to expand to accommodate pressure increases. Some fluid filters from the vascular space to the tissue compartment (blue), the amount of which is determined by the endothelial resistance and relative contributions of the vascular and interstitial colloid osmotic pressure (COP) sources. The COP values are updated each time step according to the concentration of albumin in each compartment. Each tissue compartment contains an intracellular space that predominantly regulates ion flux. Blood volume is maintained by returning filtered fluid to the vena cava via a rudimentary lymphatic system. The lymph pump is necessary because the interstitial space has lower hydrostatic pressures than the vena cava. The pre- and post-lymphatic resistances are tuned so that the amount of lymph returned equals the amount of fluid filtered at steady state. Check valves are included to ensure that no lymph flows backward.

BioGears models fluid exchange between the vascular and extravascular spaces using the cardiovascular circuit introduced previously. As Figure 2 indicates, each vascular compartment maintains a link to an associated tissue compartment via a series of elements. This mapping is one-to-one except in the case of the small intestine, large intestine, and splanchnic vascular compartments, which are collectively linked to a “Gut Tissue” compartment. Each vascular-tissue link consists of two pressure sources and a resistor. The resistor signifies the permeability of the endothelium to fluid. Likewise, the pressure sources represent the vascular and interstitial colloid osmotic pressures (COP) that arise from the relative impermeability of the endothelium to large plasma proteins. We can define the volumetric flux (*J*_*V*_*)* across resistor *R*_1_ in Figure 2: as

(25) $$\begin{array}{l} J_{v}=\frac{P_{1}\;-\;P_{2}}{R_{1}}=\frac{P_{v,h}\;-\;COP_{v}\;-\;(P_{i,h}\;-\;COP_{i})}{R_{1}}\\ \;=\frac{1}{R_{1}}\left(\Delta P_{h}-\Delta COP\right). \end{array}$$

The ohmic relationship described by Equation (25) approximates Starling's Equation for capillary exchange:

(26) $$\begin{array}{l} J=L_{P}S\left(\Delta P_{h}-\sigma \Delta COP\right). \end{array}$$

This approximation is particularly good given that healthy endothelial cells exhibit a reflection coefficient (σ) near unity (Pietribiasi et al., 2016).

All BioGears tissues return fluid to a common lymph compartment via pathways comprised of a pressure source, a resistor, and a valve in series (Drake et al., 1986). This structure maintains basal lymph return to the vena cava while preventing backflow. Collectively, the tissue circuit is tuned so that each organ experiences a net zero change in extravascular fluid and the total amount of fluid filtered and returned by the lymph amounts to 4.0 L/day, within the ranges reported in literature (Boron and Boulpaep, 2017).

BioGears solves the circuit defined by Figure 2 at every time step using the SE Circuit interface to determine the amount of fluid to move across each node. The hydrostatic pressures are obtained from the previous state of the circuit, while the hydraulic resistance is a property of each tissue. We assume albumin, a BioGears substance, to be linearly correlated with total plasma protein concentration (C_pp_, g/dL) and calculate COP_i_ and COP_v_ using the Landis-Pappenhaimer relationship (Mazzoni et al., 1988):

(27) $$\begin{array}{l} COP=2.1C_{pp}+0.18C_{pp}^{2}+0.009C_{pp}^{3}. \end{array}$$

Given that Equation (27) requires an updated albumin concentration each iteration, we employ the Patlak equation to determine albumin flux between each vascular-tissue compartment pair (Rippe and Haraldsson, 1994).

(28) $$\begin{array}{lll} J_{Alb} & = & J_{V}\cdot \left(1-\sigma \right)\cdot \left(\frac{C_{Alb,p}-C_{Alb,i}\cdot e^{-Pe}}{1-e^{-Pe}}\right) \end{array}$$

(29) $$\begin{array}{lll} Pe & = & J_{v}\cdot \frac{1\;-\;\sigma }{PS} \end{array}$$

Equation (28) describes the relative contributions of convective and diffusive albumin flux to total albumin flux (*J*_*Alb*_) as a function of the Peclet number (*Pe*, Equation 29). At low fluid filtration rates (*J_V_*), *Pe* is small and the difference between the plasma albumin concentration (*C*_*Alb, p*_) and interstitial albumin concentration (*C*_*Alb, i*_) drives flux. *J*_*Alb*_ becomes proportional to the product of *J*_*V*_ and *C*_*Alb, p*_ in the limit as *J*_*V*_ grows, indicating that convective flux dominates as the rate of filtration increases. We assume a constant diffusion capacity (PS) across the entire diffusion distance for simplicity. Albumin returns to the bloodstream through the lymph system via convective transport (Pietribiasi et al., 2016), maintaining constant vascular and interstitial protein concentrations under normal conditions.

**Figure 3:**
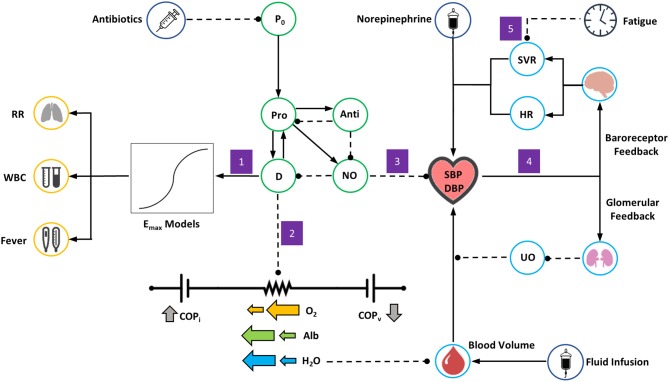
Overview of the BioGears Sepsis Model. Green circles represent elements of the acute inflammatory response model, light blue circles elements of BioGears feedback models, dark blue circles relevant treatment actions, and yellow circles other aspects of systemic inflammatory response syndrome (SIRS). Solid lines with arrows indicate up-regulation or positive effect while dashed lines indicate inhibition or negative effect. The model is triggered by an initial pathogen load (P_0_) that sets off a cascade of pro-inflammation (Pro) and anti-inflammation (Anti). As pro-inflammation becomes too severe, tissue damage (D) increases. Free radical production in the form of nitric oxide (NO) follows. The following chain of events occurs in response: (1) The presence of inflammation and tissue damage leads to symptoms of systemic inflammatory response, modeled by general E_max_ relationships. (2) As damage worsens, the resistances on all vascular-tissue endothelial pathways on the BioGears cardiovascular circuit decrease, leading to hypovolemia and hypoalbumeria and a tendency toward hypotension. (3) Nitric oxide accumulation exerts an additional hypotensive effect. (4) Hypotension induces a response from baroreceptor and glomerular feedback models. Baroreceptor feedback increases systemic vascular resistance (SVR) and heart rate (HR). Glomerular feedback increases fluid retention, causing a drop in urine output (UO) that opposes volume loss. (5) As symptoms of systemic inflammation continue unabated, the ability of baroreceptors to increase SVR becomes inhibited.

We model the deterioration of the glycocalyx under severe inflammatory conditions by decreasing the endothelial wall resistance (R_1_, Equation 25) of every vascular-tissue pair proportionately to the reduction of tissue integrity (TI, Equation 18). Doing so increases the amount of fluid transported from the bloodstream to the interstitium, as Equation (25) indicates. We also assume that the accumulation of tissue damage decreases the reflection coefficient (σ) of every BioGears tissue compartment. These alterations give rise to greatly enhanced albumin flux according to Equation (28). As the albumin concentration gradient dissipates, the vascular and interstitial colloid osmotic pressures (Equation 27) begin to converge. This development further exacerbates fluid loss (Equation 25). If this loop remains uninterrupted and tissue integrity (TI) reaches low enough levels, both hypovolemia and hypoalbuminemia will occur (Figure 3, Box 2).

The BioGears transport and feedback models produce a physiologically sound cascade of events in response to the above modifications to the cardiovascular circuit. The diminishing blood volume lowers the mean arterial pressure (MAP), a development detected by the BioGears baroreceptor model. This model increases systemic vascular resistance (SVR) and heart rate (HR) in an effort to reverse hypotension. Simultaneously, the BioGears renal system responds to hypotension by decreasing glomerular filtration rate to retain fluid (Figure 3, Box 4).

We should note that, while the literature supports the notion that all organs are susceptible to endothelial dysfunction (De Backer et al., 2014), we recognize that only a subset of organs will be affected in a given septic incident, likely over disparate time intervals. But, as stated previously, we make the assumption that the tissue integrity state variable represents global organ health. Future model iterations should investigate accumulation of tissue damage in varying organs on different time scales.

#### Microcirculatory Distress

Microcirculatory dysfunction plays a prominent role in the progression of sepsis. Research indicates that measures of microcirculatory distress correlate more strongly with patient mortality than do hemodynamic indices (Sakr et al., 2004; De Backer et al., 2013). Irregularities in endothelial integrity, red blood cell shape, cell signaling, and coagulation all contribute to microcirculatory deterioration (Ince and Mik, 2015). These alterations inhibit adequate tissue perfusion and oxygenation, contributing to organ failure (Ince and Mik, 2015).

Given that compartments represent the highest-fidelity organization of data in BioGears, we cannot mechanistically model aspects of microcirculatory dysfunction such as capillary perfusion heterogeneity and mitochondrial dysfunction. Instead, we qualitatively model the physiological endpoint of these processes: tissue hypoxia. The combination of unbalanced capillary perfusion and red blood cell deformation produces an oxygen deficit in regions of tissue, causing localized hypoxia and potentially leading to mitochondrial distress (Ince and Mik, 2015). We capture this effect by incrementing an energy deficit variable as a function of tissue integrity. We subtract this deficit from the output of the BioGears energy production and nutrient consumption model; as a result, the model detects insufficient energy levels on its next iteration and uses up more nutrients to combat it. As the deficit worsens, the production/consumption model increasingly leans on anaerobic energy production, which causes lactate accumulation in the tissue and hyperlaktemia, a critical clinical marker in sepsis diagnosis (Singer et al., 2016).

#### Systemic Vasodilation

A hallmark of sepsis is persistent hypotension (Singer et al., 2016). While circulating volume depletion undoubtedly contributes to this effect, it does not alone account for its severity. One would expect volume reduction to induce corrective action from the baroreflex in the form of vasoconstriction; yet septic patients typically exhibit drastically reduced SVR—as low as 25% of baseline (Young, 2004)—indicating instead the presence of vasodilation. The inflammatory response must therefore contribute significantly in this respect. While many mediators likely play an important role, significant research has focused on the free radical nitric oxide (NO), a potent vasodilator generated as pro-inflammatory chemicals up-regulate the activity of inducible nitric-oxide synthase (iNOS) (Vincent et al., 2000). Other investigations have noted concurrent disruption of the sympathetic arm of the baroreflex during acute infection. Animal models of endotoxemia have demonstrated that infection can cause the baroreceptor operator curve to reset (Tohyama et al., 2018) and can decrease baroreceptor sensitivity (Radaelli et al., 2013). Likewise, endotoxemic studies in humans have noted depressed responsiveness to sympathetic nervous activity, particularly in the vascular smooth muscle (Sayk et al., 2008 and Brassard et al., 2016).

We incorporate both nitric oxide-mediated hypotension and sympathetic dysfunction in our model. The BioGears PBPK/PD model accepts the systemic nitric oxide count determined by Equation 19 as an input and calculates changes in SBP and DBP (Figure 3, Box 3). This calculation employs a sigmoidal E_max_ model and thus requires us to estimate both the maximum effect of NO on blood pressure and the NO count that defines the midpoint of the sigmoid. The BioGears baroreceptor model derives from the work of Ottesen et al. (2004). This model determines the deviation of the MAP from its set-point and adjusts heart elastance, heart rate, vessel compliance, and vessel resistance accordingly. We do not attempt to model SVR depression mechanistically, but instead reduce the vessel resistance gain determined by the baroreceptor model as a function of the duration of the SIRS event (Figure 3, Box 5).

Finally, we must note that the BioGears diastolic blood pressure (DBP) tends to be less responsive than the SBP to homeostatic disruption. Given that calculations of MAP weigh diastole more heavily than systole, this issue results in the BioGears MAP being slow to respond, particularly in the early stages of hypovolemia. Consideration of the hypotensive activity of nitric oxide and of baroreceptor irregularity mitigate this issue, but it is possible that we had to overstate their severity in order to do so.

#### Other Pathophysiology

We empirically relate the AIR model to other BioGears outputs to produce heretofore unaddressed clinical markers of inflammation, namely tachypnea, fever, and altered white blood cell count (Figure 3, Box 1). We assume the latter to be directly related to the active neutrophil population (N_A_, Equation 9) by a proportionality factor that accounts for conversion to the clinically relevant units of ct/μL. The relationships describing tachypnea and fever progression are sigmoidal in nature, taking the form:

(30) $$\begin{array}{l} E=\;\frac{E_{max}{\left(1\;-\;TI\right)}^{\gamma }}{E_{50}^{\gamma }\;+\;{\left(1\;-\;TI\right)}^{\gamma }}. \end{array}$$

Each effect, E, approaches a maximum value (E_max_) as tissue integrity decreases. Estimates for E_max_ were determined according to diagnostic criteria for sepsis (Dellinger et al., 2013). Since tachypnea and fever constitute early signs of inflammation, their respective E_50_'s (the value of 1-TI at which half of the maximum effect is observed) are set fairly low. Finally, the parameter γ, which determines the steepness of response, is set to 1 for both effects.

#### Interventions

BioGears supports numerous interventions that can be applied during a simulation, the most relevant for sepsis treatment being fluid administration, vasopressors, and antibiotics. The BioGears Substance Compound Infusion class handles fluid challenges. Users define an infusion object by specifying a dose volume, a rate of infusion, and the identity of a compound. Currently defined compounds include blood, normal saline, Ringer's lactate, and albumin colloid. The compound definitions specify concentrations of their constitutive components, which the infusion model uses in conjunction with the administration rate to increment the appropriate component masses in the virtual patient.

Vasopressors, and liquid-based drugs, in general, can be introduced to a simulation using either bolus dosing or continuous infusion. Both actions require a drug name and concentration to instantiate them, in addition to either a bolus size or rate of administration. BioGears contains a detailed whole-body PBPK/PD model that governs drug absorption, distribution, metabolism, and elimination. This model uses substance-specific physiochemical data to predict tissue-plasma partition coefficients and to generate a whole-body concentration profile for a drug (Clipp et al., 2016; McDaniel et al., 2019). The BioGears renal and hepatic systems handle drug metabolism and elimination. A number of pharmacodynamic effects are modeled using equations similar to Equation 30 that depend on drug concentration and calibrated effect modifiers. For drugs known to exhibit slow receptor-binding kinetics, a delay compartment is included. The BioGears substance library currently contains three vasopressors that may be of interest for sepsis treatment scenarios: vasopressin, norepinephrine, and—to a lesser extent—epinephrine. A complete list of supported drugs, as well as a more in-depth description of the PBPK/PD model, can be found in our online documentation.

The BioGears pharmacodynamics functionality extends to antibiotics. We assume that antibiotics act solely against infection (exerting no other effects on patient physiology) according to the relationship

(31) $$\begin{array}{l} S_{net}=S_{max}-\frac{\left(S_{max}\;-\;S_{min}\right){\left(C_{u}/MIC\right)}^{\gamma }}{{\left(C_{u}/MIC\right)}^{\gamma }\;-\;S_{min}/S_{max}} \end{array}$$

(Regoes et al., 2004; Ankomah and Levin, 2014). Equation (31) defines the reduction in net bacteria growth rate (S_net_) as function of free (unbound) antibiotic concentration (C_u_), the minimum inhibitory concentration of the bacteria (MIC), the minimum growth rate imposed by the antibiotic (S_min_) and the growth rate of the bacteria in the absence of antibiotic (S_max_). An interesting result of Equation 31 is *C*_*u*_ = *MIC* → *S*_*net*_ = 0 for any and all parameter values. That is, though bacterial growth will stagnate at sub-inhibitory antibiotic levels, the bacteria population will not decrease until antibiotic concentration exceeds the minimum value. An advantage of this outcome is that we do not need to define an EC_50_ value for antibiotics; that value will be determined by the antibacterial effect S_min_ that we choose. Given that the majority of antibiotic pharmacodynamics studies are carried out *in vitro*, which can make model parameters difficult to establish *in vivo*, we consider estimating fewer parameters to be desirable. Presently the BioGears substance library maintains three antibiotics with validated pharmacokinetic profiles: intravenous ertapenem, oral moxifloxacin, and intravenous piperacillin.

#### Infection and Sepsis Action Initiation

We define a bacterial infection in BioGears using a location, a severity, and a minimum inhibitory concentration (MIC). The location is a simple string corresponding to one of the tissue spaces in the BioGears compartment hierarchy. Presently, the location has no bearing on the progression of the simulation because we assume that bacteria colonization and invasion proceeds identically for all tissues. Future iterations of this model will seek to account for the effects of organ physiology on bacteria elimination and diffusion. The infection severity takes an enumeration from the set Mild, Moderate, and Severe and maps the value to an initial pathogen load. Though subjective, we chose this implementation because the pathogen count is in arbitrary units, which could make it challenging for users to select an appropriate initial value. We include the MIC as a proxy for bacteria type and strain, which greatly expands the permutations of bacteria-antibiotic interactions that can be explored with this model.

The pathogenesis of sepsis evolves over hours and days, which leads to long simulation times even in a model such as BioGears that runs faster than real-time. We can mitigate this limitation via the data serialization protocols defined on the BioGears CDM. By simulating infections of varying severities and serializing the engine periodically, we create a library of infected patient states in XML format that can be loaded into future simulations to reduce redundancy. Thus, one could define a BioGears sepsis action that loads the state of a severely infected patient at a time shortly before sepsis onset. The existence of this library has the additional benefit of providing multiple entry points at which treatment actions can be initiated. Therefore, the effects of treatment timing can also be investigated with minimal redundancy.

### Model Simulations

#### Infection Action Comparisons

We first demonstrated the BioGears infection action at the three supported levels of severity (mild, moderate, severe), assuming the nominal parameter values in Table 2 for the AIR model. We calibrated the initial pathogen loads of these scenarios so that the mild and moderate cases resolved with pathogen elimination and returned to baseline physiology and the severe scenario resolved in progression to septic shock. We assumed the bacteria MIC to be 16.0 mg/L. Due to the length of these simulations, we periodically introduced actions for drinking water and eating meals. Discounting these nutritional actions would have confounded our results, as BioGears models the physiological consequences of dehydration and nutrient depletion. We compared the infection actions to a control scenario in which a healthy (non-infected) patient follows an identical water and meal consumption schedule.

#### Virtual Patient Variability

BioGears allows users to set patient characteristics (e.g., gender, weight, and height) and physiological baselines, such as SBP, HR, and RR These user-defined parameters, however, do not greatly affect the trajectory of infection simulations due to the deterministic nature of the AIR model. We therefore performed additional simulations of moderate infection while varying certain parameters of the AIR model to demonstrate that the BioGears sepsis model supports variability in virtual patient outcome. We chose our variable parameters from the set {*k*_*P*_*T*_*M*_*T*__, *k*_6*M*_, *x*_66_}. The first parameter in this set was selected according to the results of the global sensitivity analysis performed by Domínguez-Hüttinger et al. (2017). The latter two parameters strongly dictate the response of IL-6, the levels of which have been found in other modeling and simulation studies to be correlated with individual patient outcome (Brown et al., 2015).

#### Sepsis Treatment Scenarios

All treatment scenarios were conducted using virtual patient states generated from the nominal BioGears severe infection action. We modeled our treatment protocols after a study described in Macdonald et al. (2017, 2018). This pilot program, called the “REstricted Fluid REsuscitation in Sepsis-associated Hypotension (REFRESH) study, sought to determine whether limiting fluid administration and initiating early vasopressor therapy improves septic patient mortality compared to standard of care. Patients who qualified for enrollment in REFRESH (suspected infection accompanied by persistent SBP < 100 mmHg after fluid challenge) were randomly assigned to either a “standard” treatment group or a reduced fluids and early pressor treatment group.

We constructed the BioGears versions of these treatment regimens as follows. Just prior to septic shock onset, we administered two separate 500 mL boluses over the course of an hour. After confirming that fluid challenge did not restore SBP, we defined a control scenario and an experimental scenario, initializing both from this septic patient state. Each scenario began with 4.5 g piperacillin/tazobactam administered intravenously over 30 min. We selected this dose based on recommendations from subject matter experts at the University of Washington with whom we collaborated. We assumed all the antibacterial activity of this combination was attributable to piperacillin and so only tracked its pharmacokinetic (PK) qualities. Furthermore, we compared the piperacillin PK profile predicted by BioGears with that established in the literature (Sörgel and Kinzig, 1993). Concurrent with antibiotic initiation, the control patient received a second 1,000 mL fluid bolus, while the experimental patient began maintenance fluids and norepinephrine at respective rates of 1 mL/kg/h and 0.18 μg/kg/min. We established that this norepinephrine dose, which falls within the range of doses investigated in the sepsis literature (De Backer et al., 2003; Beale et al., 2004), achieved the targeted MAP in the BioGears septic patient. We elected to maintain norepinephrine at a constant rate throughout this trial, but future investigations could simulate the effects of titrating it up or down over the course of treatment.

We advanced the control scenario in 30-min increments for six h, checking SBP, MAP, and UO after each run. As per the REFRESH study, we introduced a 500 mL bolus every half-hour that SBP or MAP fell below the goals defined above. The control strategy allowed for administration of an additional 500 mL saline bolus upon hourly reassessment of resuscitation status. We used UO as our reassessment marker and chose 0.5 mL/kg/h (0.625 mL/min for the standard BioGears patient) as our threshold for further bolus dosing (Rhodes et al., 2017). Thus, if we observed UO < 0.625 mL/min at the end of an hour of treatment, we provided the supplemental bolus. If after any 30-min period SPB and MAP exceeded 90 mmHg and 65 mmHg, respectively, we discontinued the protocol and initiated maintenance fluids at 75 mL/h.

We advanced the BioGears experimental treatment scenario in 1-hour increments for 6 h following background saline and norepinephrine initiation. We again used UO < 0.625 mL/min as our reassessment marker. However, in the experimental group, the supplemental bolus was 250 mL. As above, if SBP and MAP met resuscitation goals after an hour, we stopped all aspects of the experimental protocol except for maintenance fluids.

It is well-established that delays in treatment initiation have drastic implementations for patient mortality in sepsis cases. The probability of survival decreases between 7 and 8% each hour that antibiotic administration is delayed after hypotension (MAP < 65 mmHg) onset (Kumar et al., 2006). A more recent study confirmed this association between delayed antibiotic administration and mortality and further found no such association with respect to time of initial fluid bolus administration (Seymour et al., 2017). We investigate treatment delays in our model by repeating the control scenario described above but deferring its initiation by 12 h.

## Results

### Infection Action Comparisons

**Figure 4:**
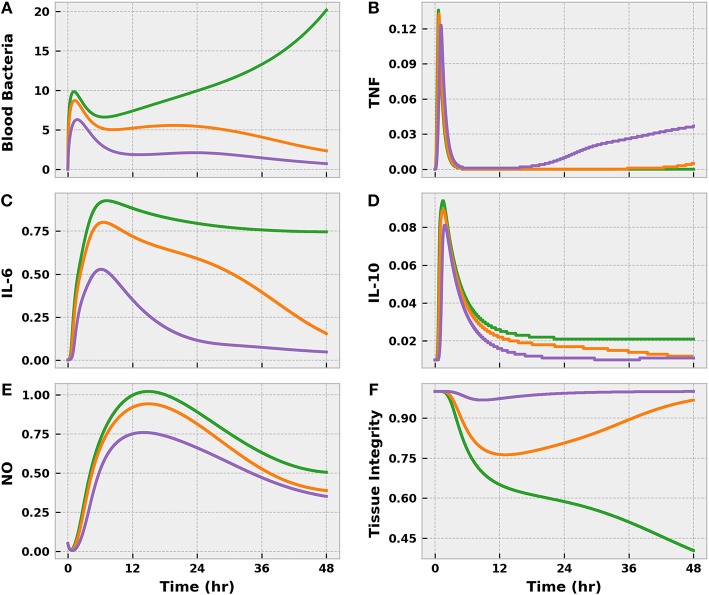
The progression of the acute inflammatory response model when the BioGears Infection action is simulated for 48 h at the three different severities: mild (purple), moderate (orange), and severe (green). These Infection action levels correspond to initial tissue bacteria counts of 1e6, 5e6, and 1e7, respectively. Selected outputs are **(A)** blood bacteria population, **(B)** TNF count, **(C)** IL-6 count, **(D)** IL-10 count, **(E)** NO count, **(F)** and Tissue Integrity. All units are in arbitrary concentration except for Tissue Integrity, which is dimensionless. Mild and Moderate infections demonstrate some infiltration of bacteria to the blood stream, but the levels are brought under control by the action of neutrophils recruited by inflammatory mediators IL-6 and TNF. Overall, the system exhibits an appropriate balance between pro- and anti-inflammatory (IL-10) activities with minor implications for tissue health. In the severe case, the bacteria population in the blood grows unchecked and pro- and anti-inflammation become imbalanced, leading to excessive and prolonged inflammation. When pro-inflammatory activity is not curtailed, the tissue accrues additional damage.

The three levels of infection action produced drastically different outcomes, as expected. In all three cases, some bacteria invaded the bloodstream from the infection site (Figure 4), but the effectiveness of the immune response in curbing bacterial growth in the blood varied. The mild and moderate infections were brought under control within 24 h post-infection, if we define “control” as sustained negative net bacterial growth. However, the moderate infection invoked a stronger response from the immune system, as indicated by the higher levels of pro-inflammatory mediators (TNF and IL-6) compared to the mild case. These inflammatory mediators were pushed to even higher levels by the severe infection, but this response was not strong enough to eliminate the blood-born bacteria. In fact, as the severe infection grew unabated, the body entered a state of excessive inflammation that the anti-inflammatory response (IL-10) could not balance. Unchecked and sustained pro-inflammation caused tissue damage to accumulate at levels not observed in the mild and moderate cases.

**Figure 5:**
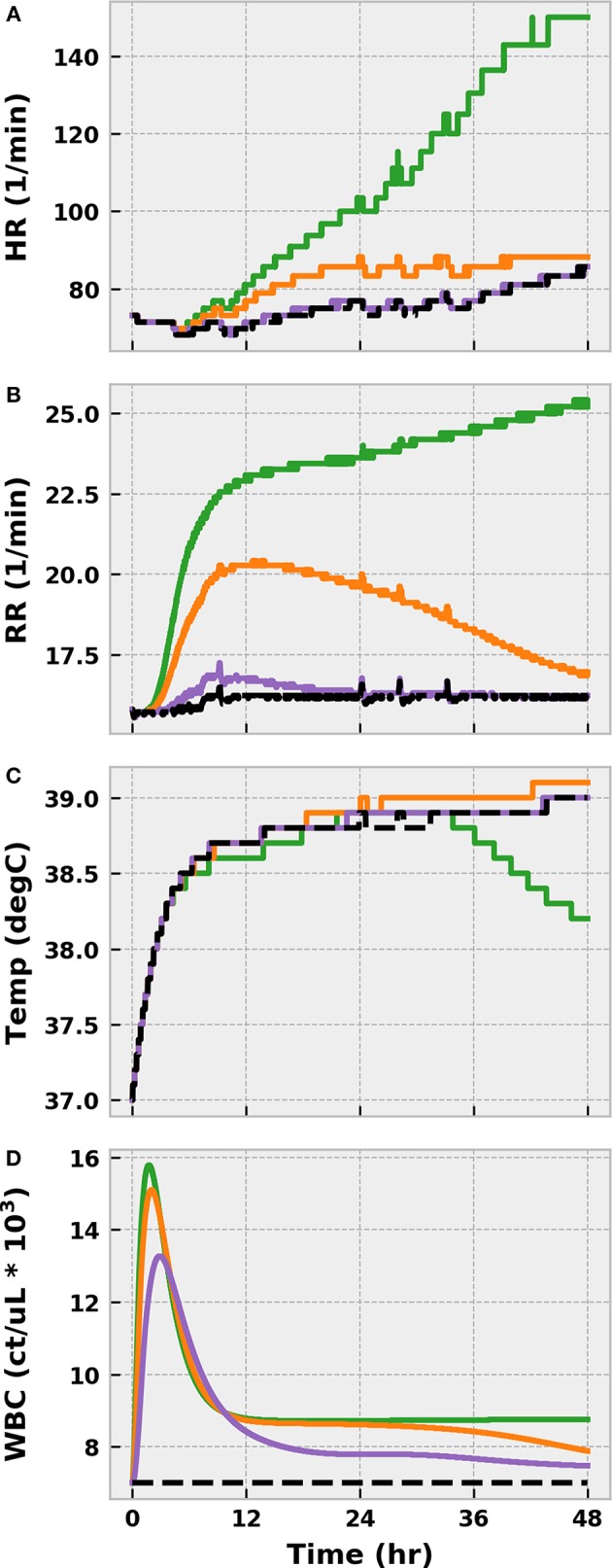
The progression of physiological markers of inflammation when the BioGears Infection action is simulated for 48 h at three different severities: mild (purple), moderate (orange), and severe (green). **(A)** HR, **(B)**, RR, **(C)** temperature, and **(D)** white blood cell count (WBC) are compared against a no-infection baseline simulation in which the same water and meal consumption schedule is followed (Black). The combination of tachycardia, tachypnea, fever, and leukocytosis in the Severe case all indicate Systemic Inflammatory Response Syndrome (SIRS). Symptoms in Mild and Moderate cases show minor deviations from baseline physiology that, for the most part, resolve over 48 h. The exception is core temperature, which increases across all scenarios and decreases more rapidly in the presence of severe infection. This outcome outcome likely indicates a minor setpoint issue in the BioGears temperature regulation model. The late decline in the severe case might be attributable to decreased blood flow to the skin, which is a factor in temperature conductance between the core and the environment in BioGears.

**Figure 6:**
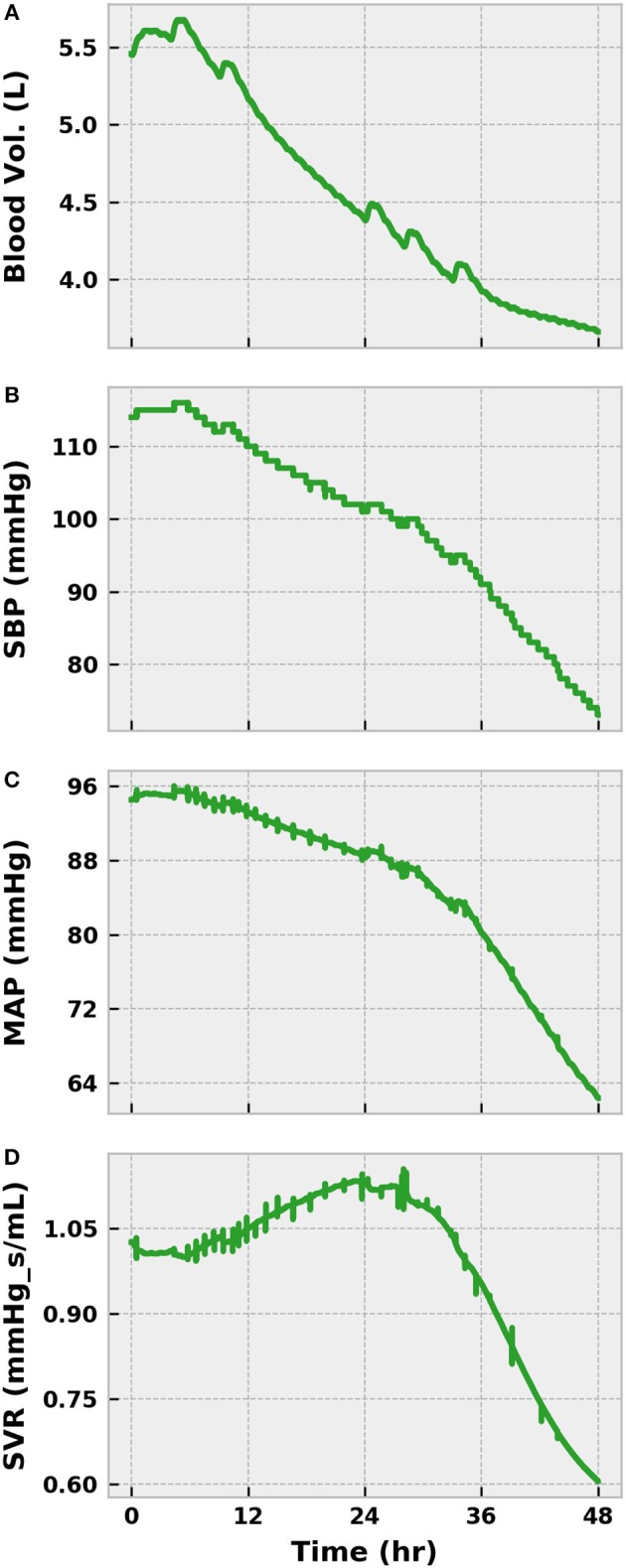
The progression of a severe infection to sepsis and septic shock in the BioGears Engine (Part 1). The accumulation of tissue damage leads to increased fluid conductance between the vascular and tissue spaces, causing a reduction in blood volume **(A)**. Feedback from the baroreceptors prevents significant loss of SBP **(B)** and MAP **(C)**, for a time by increasing SVR **(D)** and HR (Figure 5). However, sympathetic inhibition becomes noticeable after 24 h and causes SVR to decrease substantially, resulting in sharp declines in blood pressure. HR continues to increase in an effort to maintain cardiac output (CO). Using SBP < 100 mmHg and tachypnea (Figure 5) as indicators of sepsis, the virtual patient becomes septic at ~30 h.

The virtual patient exhibited clinical symptoms of infection, such as elevated tachycardia, tachypnea, fever, and leukocytosis, shortly after bacterial translocation to the bloodstream in all scenarios (Figure 5). With the exception of fever, symptoms were less pronounced in the mild and moderate cases and resolved themselves as the infection was eliminated. Conversely, the severe case progressed to a state of systemic inflammatory response syndrome (SIRS) as a result of persistent bacterial growth. Compounding matters, inflammation-mediated endothelial dysfunction had developed sufficiently by this point to induce noticeable vascular volume loss (Figure 6). The baroreflex initially combatted this volume loss by inducing tachycardia and increasing systemic vascular resistance (SVR), thereby delaying the onset of hypotension. As circulating volume continued to decline, however, these corrective efforts could not prevent SBP from dropping below 100 mmHg at 26 h post-infection. A clinical diagnosis of sepsis could be made at this time according to the Sepsis-3 definitions (Singer et al., 2016).

**Figure 7:**
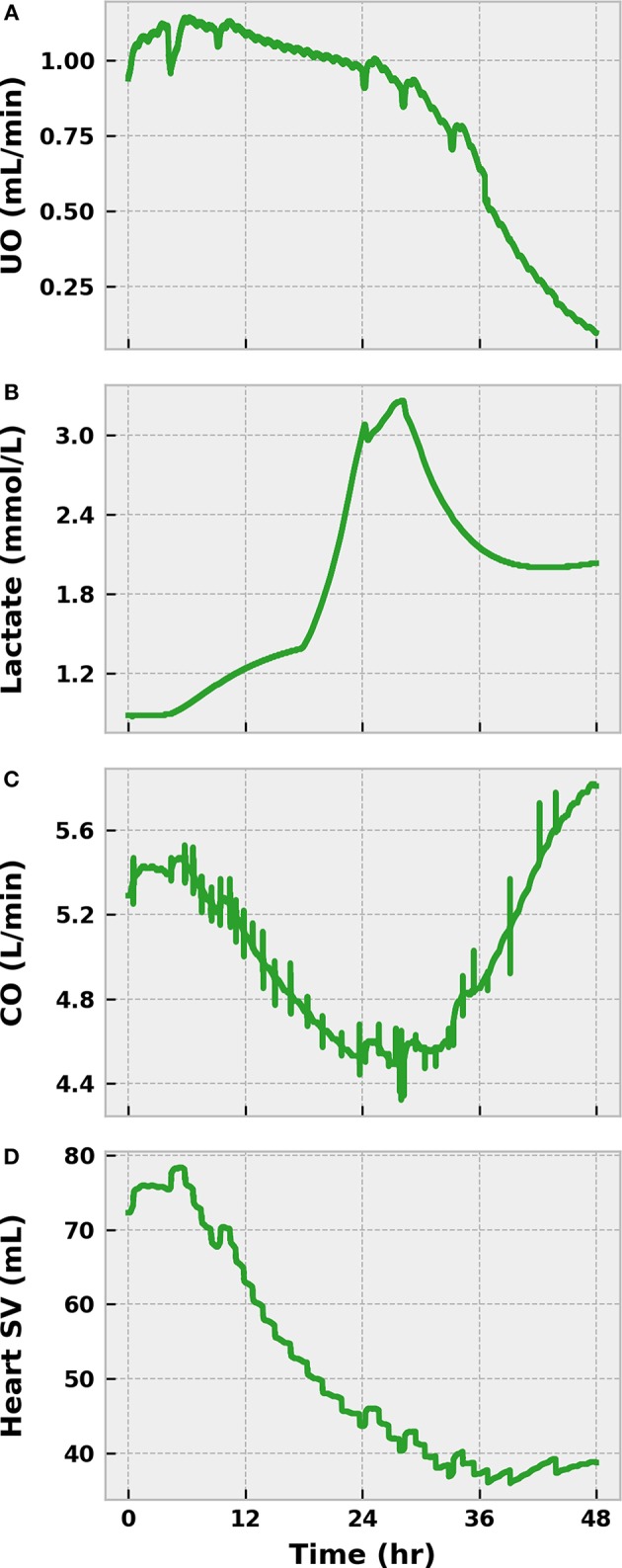
The progression of severe infection to sepsis and septic shock in the BioGears Engine (Part 2). Tubulerglomerular feedback from the renal system responds to low circulating volume by decreasing UO **(A)**. The effect of tissue damage on cellular respiration becomes apparent as lactate accumulates in the blood **(B)**. The sharp changes in concentration likely occur as the BioGears renal model attempts to consolidate substance clearance with low glomerular filtration rates. Symptoms of cardiac distress arise as **(C)** CO and stroke volume (SV) fall **(D)**. Though CO increases in the latter stages due to enhanced tachycardia, the underlying SV dysfunction remains. The virtual patient can be diagnosed with septic shock at ~45 h due to the severe hypotension (Figure 6) and the hyperlaktemia.

We declined to initiate any treatments during this initial severe infection simulation. As such, the state of the virtual patient worsened dramatically. The sympathetic arm of the baroreflex (as indicated by SVR) began to lose its effectiveness after sepsis onset as a result of nitric oxide accumulation and baroreceptor fatigue (Figure 6). UO dropped below 0.625 mL/min in the same time frame, indicating the presence of hypovolemia (Figure 7). The presence of metabolic abnormalities could be inferred after 20 h as evidenced by serum lactate levels above 2 mM (Figure 7). We considered this degree of hyperlaktemia, combined with MAP < 65 mmHg at 45 h (Figure 6), to constitute a state of septic shock. With shock unresolved, the patient entered the doorstep of cardiovascular collapse. Systemic vascular resistance approached 60% of its baseline, mean arterial pressure continued to decline, and heart stroke volume fell (Figure 7).

### Virtual Patient Variability

**Figure 8:**
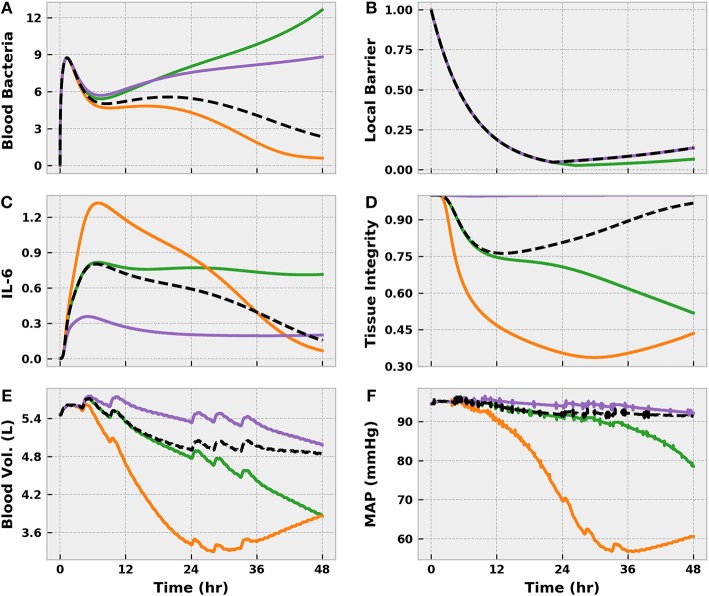
The virtual patients subsets generated by varying acute inflammatory response model parameters in the set {*k*_*P*_*T*_*M*_*T*__, *k*_6*M*_, *x*_66_} and applying a Moderate level BioGears infection action. In the nominal case (Black), pathogen is eliminated **(A)** and pro-inflammatory mediated tissue damage is minor **(D)**, as evidenced by slight fluid shift **(E)** and minimal MAP change **(F)**. Decreasing φ_*PM*_ creates a “Susceptible” population (green), which allows bacteria buildup at the site of infection and prolongs activation of the TLR switch. Enhanced TLR activity favors local barrier degradation **(B)** to promote neutrophil migration to the infection site, but this action also promotes excessive bacteria diffusion to the bloodstream, leading to sepsis. Increasing *k*_6*M*_ and *x*_66_ generates a “Hyperinflammatory” response (orange), in which the bacteria is eliminated, but interleukin-6 levels remain elevated **(C)**. This imbalanced pro-inflammation leads to tissue damage (i.e., endothelial barrier damage) that causes vascular volume shift. Finally, decreasing *k*_6*M*_ and *x*_66_ creates an “Immunosuppressed” patient (purple). Interleukin-6 levels remain low, characterizing a pro-inflammatory phase insufficient to eliminate bacteria in the blood. Though tissue damage does not accumulate, the patient will no doubt experience consequences of systemic infection.

Our parameter variability investigation revealed three subpopulations of potential interest, which we compared to the nominal model response (Figure 8). The first, which we will call the “Susceptible” population, was characterized by rapid bacteremia and sepsis onset when challenged with a moderate level of infection. We effected this response by decreasing the rate of phagocytosis by tissue macrophages (*k*_*P*_*T*_*M*_*T*__). Inhibiting phagocytosis, as one would expect, burdened the immune system by promoting bacterial accumulation in the tissue region, resulting in a steeper diffusion gradient directed toward the blood. A second, and noteworthy, effect of the slower rate of bacterial elimination was that the TLR switch remained active for longer. That is, to facilitate further neutrophil recruitment, the local tissue barrier integrity was exposed to a greater degree of degradation, creating a larger window of time in which bacterial diffusion to the blood was favored.

The other two subpopulations that we identified derived from aberrant IL-6 activity as dictated by the parameters *k*_6*M*_ (IL-6 recruitment by macrophages) and *x*_66_ (IL-6 self-inhibition). Decreasing either (or both) of these parameters produced an “Immuno-suppressed” state in which relatively low levels of blood bacteria were not eliminated. In our model, IL-6 plays a critical role in upregulating blood macrophage and neutrophil activity. Limiting IL-6 activation or exaggerating its anti-inflammatory effect thus prevented adequate immune activation. The unchecked growth in blood bacteria would be expected to produce significant pathophysiology in reality, but our model in its current iteration did not capture it because of the strong assumed correlation between IL-6 and tissue integrity (Equation 18). Conversely, increasing *k*_6*M*_ and *x*_66_ created a “Hyperinflammatory” state in which the bacteria was eradicated but the body incurred significant tissue damage due to prolonged and inadequately balanced pro-inflammation.

### Sepsis Treatment Scenarios

**Figure 9:**
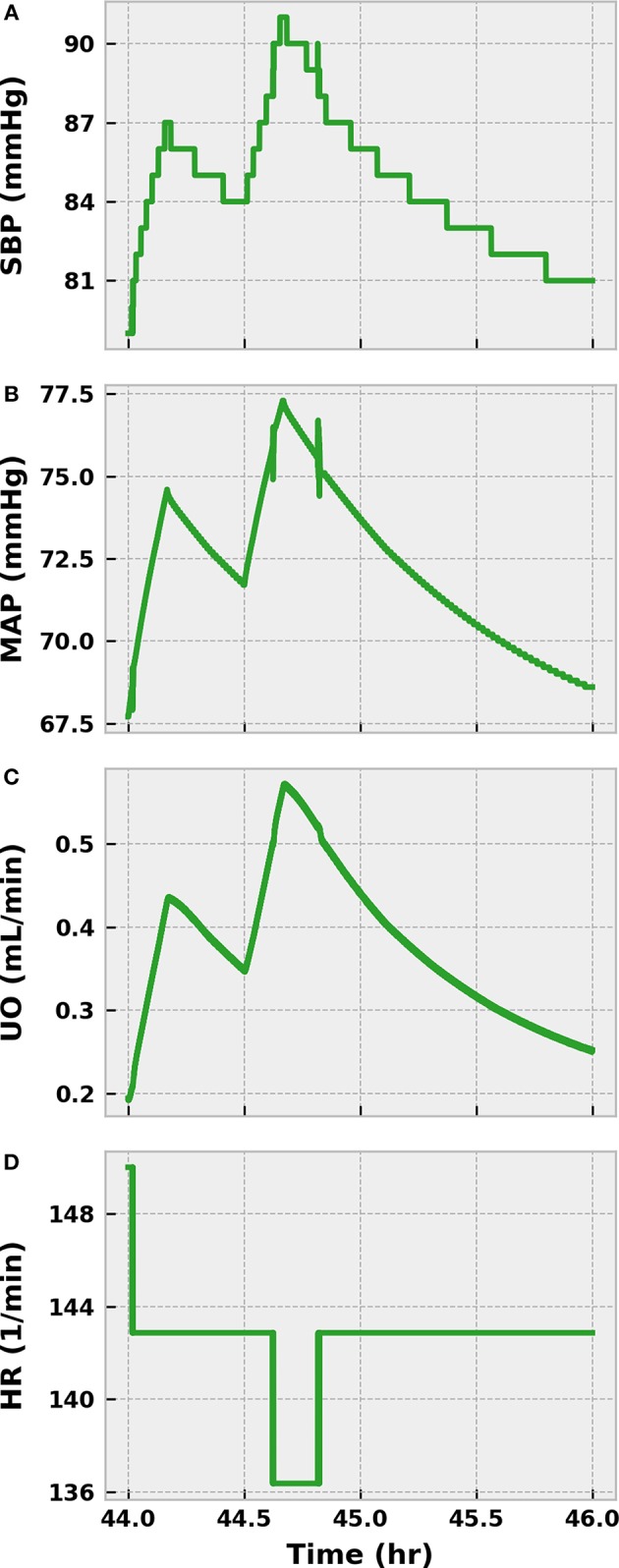
The response of the virtual patient to 1,000 mL of saline infused in two separate 500 mL boluses at 44 and 44.5 h post-infection. SBP **(A)** and MAP **(B)** transiently increase after fluid administration, but decline over the subsequent hour of observation. SBP quickly falls below 90 mmHg, the target resuscitation value in the REFRESH protocol. MAP trends downward and approaches 65 mmHg, which would characterize severe hypotension. UO shows a similarly shortlived improvement after fluid dosing **(C)** and remains below 0.625 mL/min, our threshold for hypovolemia. The improvement in blood pressure reduces some of the burden on the baroreflex, causing a small decrease in HR **(D)**. As the virtual patient is exhibiting fluid resistant hypotension, the patient can be said to be in a state of septic shock.

We administered 1,000 mL of saline over an hour beginning at 44 h post-infection, or just before our virtual patient entered septic shock (MAP < 65 mmHg). We then monitored the virtual patient for an additional hour to confirm that the fluids did not reverse hypotension. As Figure 9 shows, SBP and MAP only transiently increased. SBP remained below the threshold for establishing sepsis (100 mmHg), and MAP trended toward 65 mmHg, indicating imminent progression to septic shock. UO likewise improved temporarily only to regress, and the value of 0.25 mL/min demonstrated that the patient was experiencing severe hypovolemia. A slight improvement in heart rate was observed, but the patient remained in a state of elevated sympathetic outflow. We thus concluded that our virtual patient would hypothetically qualify for the REFRESH study.

**Figure 10:**
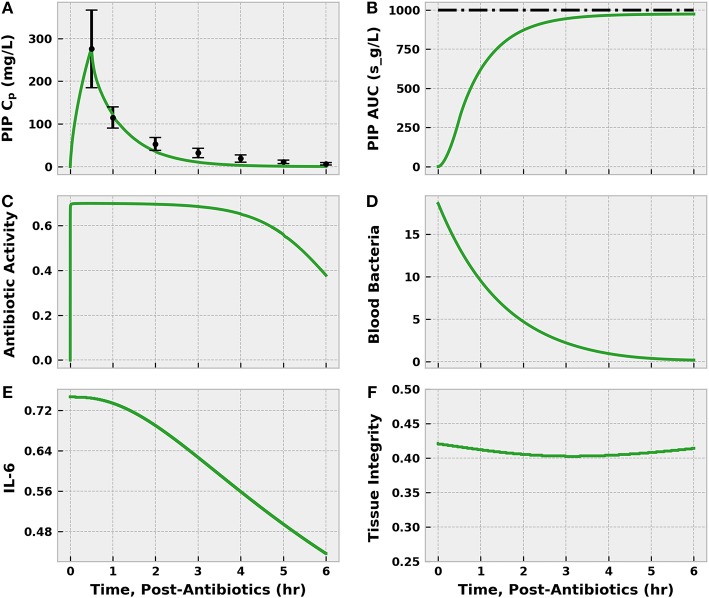
The effect of infusing 4.5 g piperacillin/tazobactam (PIP) over 30 min to the septic patient. The infusion began at 46 h post-infection (i.e., after initial fluid challenge), which we define as 0 h post-antibiotics. The plasma concentration (C_P_) estimated by the BioGears Pharmacokinetic model (green line) agrees closely with the values reported in Sörgel and Kinzig (1993) (black points) **(A)**. Error bars were estimated from individual subject data points plotted by Sörgel and Kinzig (1993). Further validation of the timing of piperacillin absorption and elimination predicted by BioGears can be seen in the calculated (green) and observed (black) area under the curve (AUC) **(B)**. Sörgel and Kinzig (1993) reported a value of 278 mg-h/L, which is ~1,000 s-g/L. The antibiotic activity parameter **(C)**, which reduces the bacteria growth rate, is a function of the unbound piperacillin plasma concentration and the MIC of the bacteria (16.0 mg/L in this simulation). Piperacillin exerts its maximal antibiotic effect (estimated at 0.70/h in our model) for ~3 h as the plasma concentration remains well above the MIC. As a result, the blood bacteria population **(D)** declines over the 6 h of observation. Reduction in bacteria count helps resolve excessive pro-inflammation and suppress IL-6 activation **(E)**. The decrease in pro-inflammation leads to improvements in tissue integrity **(F)**.

Piperacillin/tazobactam administration was the first action applied in both arms of the simulated REFRESH protocol. The BioGears pharmacokinetic model accurately characterized the plasma profile of piperacillin in terms of concentration and area under the curve (Figure 10). The antibiotic pharmacodynamics model predicted that, assuming an MIC of 16.0 mg/L, piperacillin would exert its maximum antibacterial effect (a tuned value) for ~3 h. As a result, the bacteria count in the blood decreased substantially over the 6 h of treatment. The sustained bacterial elimination that occurred even as the antibacterial effect of piperacillin diminished likely was mediated by blood neutrophils. The population of IL-6 began to decrease as bacterial levels declined, directly leading to improvements in tissue integrity.

**Figure 11:**
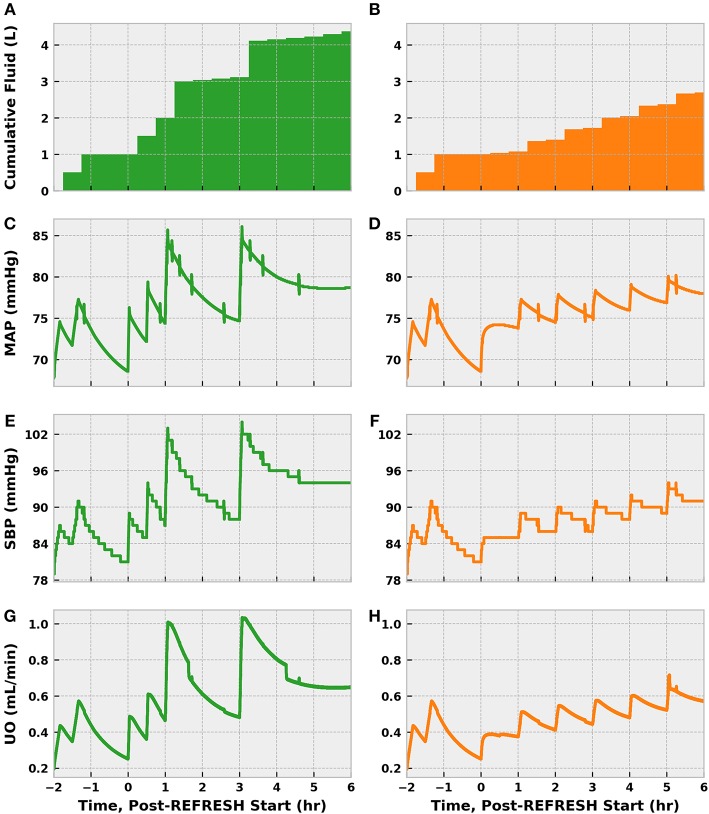
The progression of treatment and reassessment metrics in simulations following the REFRESH control (green, left) and experimental (orange, right) treatment protocols. We denote time = 0 h as the start of REFRESH protocol and time < 0 corresponds to the period of initial fluid challenge and observation (see Figure 9). Hours −2 to 0 are identical for each patient, since we applied the same bolus dose schedule prior to REFRESH initiation. We see that the cumulative amount of fluid administered over the eight total treatment hours is 4.25 L for the control patient **(A)** and 2.75 L for the experimental patient **(B)**. The control patient also experienced greater swings in pressure **(C,E)** and volume status **(G)** due to the larger fluid boluses administered (500 vs. 250 mL). The effect of early norepinephrine therapy to the treatment patient is evident in the increase in MAP **(D)** and SBP **(F)** at 46 h. The treatment patient received multiple 250 mL boluses to address persistent hypovolemia, as indicated by UO **(H)** below the target of 0.625 mL/min (0.5 mL/kg/h). Both patients reach the target SBP of 90 mmHg.

Cumulative fluid (including the preliminary bolus) administered in the control scenario outpaced that in the experimental scenario 4.25–2.75 L (Figure 11). Each fluid challenge can be clearly seen in the spikes in the plots of MAP, SBP, and UO. The more gentle increase in MAP and SBP in the experimental scenario shortly after 0 h stems from the norepinephrine infusion. Both simulated patients exceeded the MAP goal of 65–70 mmHg early in treatment but did not exhibit sustained SBP > 90 until ~ 4.5 (control) and 5.5 (experimental) hours post-treatment initiation. The experimental patient remained hypovolemic longer than the control patient, as indicated by UO.

**Figure 12:**
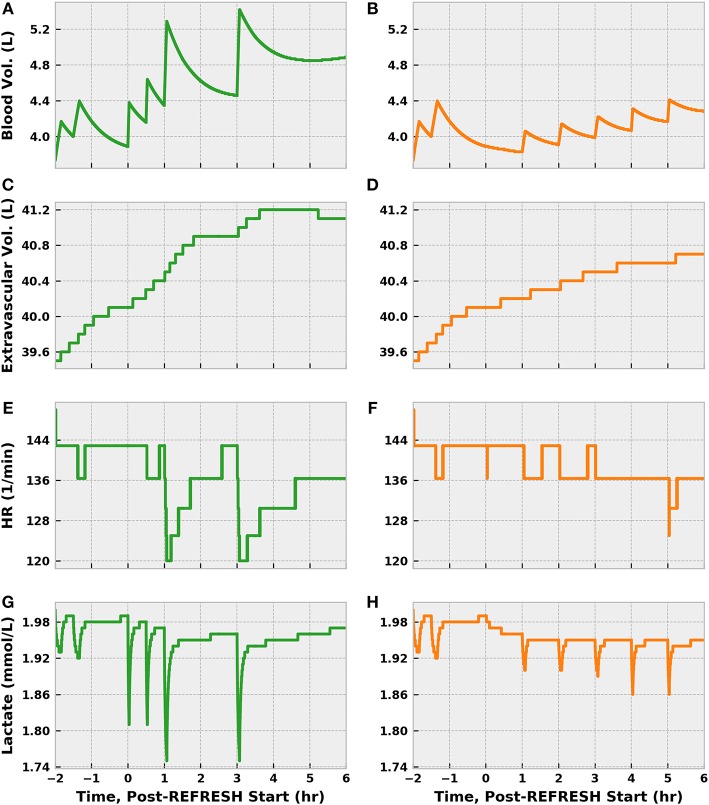
The progression of other physiological indices in simulations following the REFRESH control (green) and experimental (orange) treatment protocols, following the same time conventions. The more aggressive fluid infusion schedule followed in the control case sees faster restoration of blood volume **(A)** compared to the experimental case **(B)**. However, the control patient loses more fluid to the extravascular space **(C)** than does the experimental patient **(D)**. Tachycardia remains present in both instances **(E,F)** but shows a declining trend as blood volume is restored. Lactate levels remain elevated as well **(G,H)**, though some improvement can be observed.

Though receiving 1.5 L more fluid, the control patient exhibited a blood volume only 600 mL greater than the experimental patient after 8 cumulative treatment hours (Figure 12). Some of this difference can be accounted for by differences in UO, but fluid loss to the extravascular space contributed significantly as well. In fact, the control patient lost about 400 mL more fluid from the vasculature than did the experimental patient. The patients saw nearly identical improvements in heart rate, which is sensible given the similarity in MAP at 6 h, but remained tachycardic. Reductions in lactate molarity were also marginal.

**Figure 13:**
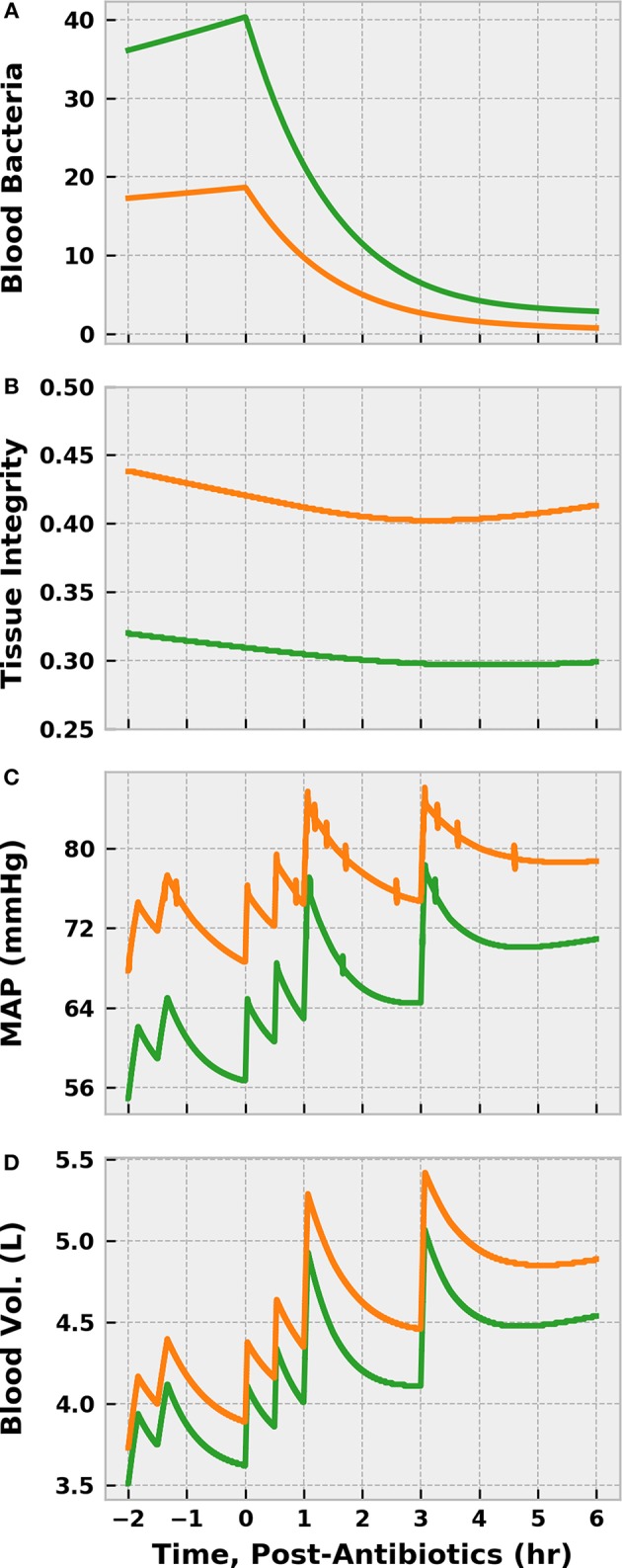
The effect of delaying antibiotic treatment in simulations of sepsis treatment. We compare the control REFRESH virtual patient described previously (orange) to another virtual patient subjected to the same treatment regimen after a delay in treatment initiation of 12 h (green). We denote time = 0 h as the time of antibiotic administration (and REFRESH start) and *t* < 0 corresponds to initial fluid challenge and observation (see Figure 9). Time 0 for the control patient corresponds to 46 h post-infection, while time 0 for the delayed treatment patient corresponds to 58 h post-infection. We see that the blood bacteria population ~ doubled as a result of the delay in antibiotic administration **(A)**. This prolonged period of heightened inflammation causes a further reduction in tissue integrity **(B)**, allowing more vascular volume loss **(D)** that contributes to more severe hypotension **(C)**. Though the antibiotics eliminate the bacteria, the tissue integrity is slower to begin recovery in the case of delayed treatment. Furthermore, fluid resuscitation is not as effective in restoring vascular volume and arterial pressure.

Predictably, delaying antibiotic administration for 12 h allowed the bacterial population in the blood to ~ double (Figure 13). Though piperacillin was still able to reduce the bacteria count, the extended period of inflammation resulted in a large reduction in tissue integrity prior to treatment onset. The resulting increase in endothelial permeability allowed additional vascular volume loss and exacerbated blood pressure depression. As such, a fluid infusion protocol identical to the control scenario above did not restore blood volume or arterial pressure to the same degree. Furthermore, the delayed-treatment patient remained in a state of hypotension for far longer than the control, which could have deleterious consequences.

## Discussion

The BioGears infection model captured important clinicals markers in the evolution of systemic inflammatory response and, in extreme cases, the progression to sepsis. The underlying dynamic model of inflammation demonstrated graduated responses to bacterial innocula of varying sizes, leading to early signs of infection, such as tachypnea, leukocytosis, and fever, on a realistic time-scale. While the model opposed a degree of bacteremia via recruitment of phagocytotic agents, promoting recovery from infection, sufficiently large infections lead to unchecked bacterial colonization in the bloodstream. In this event, the aforementioned symptoms worsened and tachycardia developed as sustained inflammation disrupted blood pressure homeostasis. Systemic and prolonged inflammation induced deterioration of the vascular endothelium that caused fluid shift and relative hypovolemia. Concurrently, nitric oxide, a byproduct of the inflammatory response, exerted a vasodilatory effect that opposed the sympathetic activity of the baroreflex. This reflex eventually fatigued to the point that elevated systemic resistance could not be maintained, and the virtual patient became extremely hypotensive and entered septic shock.

The infrastructure of the BioGears physiology engine and the range of actions its supports make feasible the development of complex sepsis intervention scenarios. We demonstrated that applying fluid bolus actions produced transient increases in blood volume, MAP, and UO, the magnitude of which depended on the length of time over which the patient had been septic. The BioGears drug administration actions allowed us to investigate norepinephrine and piperacillin infusions. The former increased MAP and helped to maintain normotension, while the latter eliminated bacteria, thereby curbing excessive pro-inflammation and promoting recovery of tissue integrity. That these drug effects exhibited physiologically reasonable onset and offset times is attributable to the accuracy with which the BioGears whole-body PK/PD model predicted their respective plasma concentration profiles.

Of critical importance to the usefulness of this model, virtual patient outcomes differed according to model parameterization and the timing and types of actions applied. We showed that distinct inflammatory response trajectories can be generated from the same level of infection by varying a small subset of model parameters. A notable phenotype we identified demonstrated severe hypotension in spite of infection resolution due to inadequate anti-inflammatory activation. Our simulation of the two REFRESH protocols produced patients who differed in volume and blood pressure status after multiple hours of treatment. We would not use our results to favor one protocol over the other. However, we could make observations, such as the smaller fluid boluses administered in the experimental case helped the patient avoid the more drastic shifts in blood pressure experienced by the control. We might also observe that these smaller fluid boluses kept the experimental patient in a hypovolemic state longer than the control patient, potentially mitigating the positive effects of curtailing blood pressure fluctuations. Or, we could note that, given the larger volume of fluid administered and the expanded extravascular volume, the control patient could be at increased risk for reperfusion injury compared to the experimental patient. Finally, our model was sensitive to treatment timing. Delaying antibiotics by 12 h worsened virtual patient hypotension and, though the patient still responded to treatment, recovery was prolonged.

In light of the above, the BioGears sepsis model has potential applicability to both identification and management of sepsis. Accurate predictive modeling that accounts for multiple physiologic inputs and host comorbidities could greatly improve risk stratification systems early in the identification of sepsis and could also reveal which factors contribute most to the progression of the disease. From a management standpoint, much remains to be learned. Despite multiple clinical studies, research into the optimization of fluid balance and vasopressors continues. Certain medications, such as steroids and statins, have not been shown to be universally beneficial in sepsis outcomes. However, specific patient populations may benefit on an individual basis, and BioGears could contribute to this understanding without the complexities of large human clinical trials. More novel therapies, such as angiotensin II, vitamin C, and thiamine, have shown potential benefit in small studies, but their clinical utility remains uncertain. This sepsis model, supported by the BioGears substance definition interface, could shed light on the effects of these therapies and the manner in which physiologic parameters influence them. A further application of this model could be to improve the training of caregivers managing septic patients. Simulated patient scenarios derived from BioGears modeling could enhance training in the recognition of early sepsis markers and in the management of sepsis.

There are some limitations of the current iteration of this model that should be addressed to enhance its applicability. The BioGears sepsis model primarily focuses on the cardiovascular response to systemic infection. As such, we do not presently consider the potential development of acute lung injury (ALI) or acute respiratory distress syndrome (ARDS) secondary to sepsis. Inclusion of these complications in the next version of our model is a reachable goal given the maturity of the BioGears respiratory model. Like the cardiovascular model, the BioGears respiratory model implements a fluid circuit that calculates fluid flow and substance transport. The cardiovascular and respiratory models communicate via a diffusion method that determines oxygen and carbon dioxide transport as a function of alveolar surface area and pressure gradient. By decreasing the lung compliances on the respiratory circuit and reducing the surface area available for diffusion, we could likely effect impaired oxygen uptake. These actions would ideally produce outputs consistent with ALI and ARDS, like reduced P_a_O_2_/FiO_2_ ratios. Furthermore, BioGears maintains a ventilator model, so addressing lung injury would open additional treatment modeling opportunities.

Another aspect of sepsis not completely addressed by our model is coagulopathy. Disruptions in the coagulation pathway occur in most instances of sepsis and therefore likely contribute significantly to organ dysfunction (Simmons and Pittet, 2015). Additionally, the coagulation and inflammation cascades are tightly interwoven. Future iterations of our model should, at minimum, take an approach similar to Kumar (2004) and introduce coagulation agents such as tissue factor, thrombin, and activated protein C, and their associated interactions, to the acute inflammation system of ODEs. A better, albeit longer-term, approach would be to enhance the fidelity of the BioGears blood chemistry model. Some work has already begun to this effect, as we are in the process of expanding our whole blood substance definition to include AB antigens and antibodies for the purpose of modeling blood transfusions. We could introduce similar component definitions for key mediators in both the coagulation and inflammation pathways. This effort would involve transitioning the state variables in the acute inflammatory model to BioGears substance data types, which in turn would require obtaining validated synthesis, distribution, and elimination data for each mediator. This more rigorous strategy would effectively embed the inflammation model within the BioGears compartment hierarchy, an advantageous outcome for a number of reasons. We would have the ability to specify exact locations of infections and more accurately model bacteria transport to and circulation through the bloodstream. The fidelity of our antibiotic model would then improve, as we would be able to account for drug-pathogen interactions on a compartment level. Finally, simulations of experimental therapies like activated protein C would proceed more mechanistically rather than relying on phenomenological relationships.

While we demonstrated that our model could perform simulations personalized to specific patient information, we must note that doing so required interaction with the BioGears source code. The BioGears API does include a patient definition interface; however, though it currently supports personalization of characteristics such as age, gender, height, and weight and physiological baselines such as SBP, RR, and metabolic rate, it does not allow manipulation of the inflammation model. We will thus work to validate our model over a wide range of parameters so that we can expose key inflammation parameters to the API, giving users the ability to define virtual patients with unique responses to infection. This effort will be part of our larger push to improve the BioGears user experience. We will shortly begin development of a graphical user interface (GUI) that will contain a library of pre-defined simulation scenarios and modules for scenario building and output visualization. We hope that this GUI will lower the barrier of entry to working with BioGears.

Finally, we are making progress on various improvements to the overall fidelity of the BioGears Engine which, though not specific to the sepsis model, will enhance it nonetheless. For instance, we just begun to restructure the BioGears nervous system so that it consolidates feedback from various systems into a unified signal rather than reacting to inputs individually. Part of this work will involve revisiting the current baroreceptor model and the manner in which that model interacts with substances that affect SVR, which has clear implications for how our sepsis model responds to nitric oxide accumulation and norepinephrine administration. Longer-term, we hope to increase the resolution of our models from the tissue level to the cellular level to more accurately capture biochemical activity. We have already made some progress in this regard with a model of intracellular volume regulation via active ion exchange. Conceivably, we could craft a similar representation of respiration at the cellular level, which would allow us to better model mitochondrial dysfunction during sepsis. We could then approach localized hypoxia and lactate production and accumulation far more mechanistically. These projects underscore both the breadth and depth of the physiology that can be modeled with BioGears and speak to its potential relevance across a wide range of *in silico* clinical studies.

## Conclusion

By leveraging the BioGears framework, we have developed a mathematical model that forges a link between pathogen-induced inflammatory dysfunction and sepsis. The model captures the clinical mileposts used by physicians to describe sepsis progression, including systemic inflammatory response, severe sepsis, and fluid-resistant sepsis (septic shock). Furthermore, the treatment protocols modeled show distinct patient outcomes, indicating that the model is sensitive to intervention strategy and timing. These features demonstrate the usefulness of this model as a trainer for medical staff. With additional refinement, this model could become a powerful tool for investigating the efficacy of new therapies and uncovering heretofore unknown sepsis features.

## Data Availability Statement

The code used to perform these simulations can be run without modification (with the exception of the patient variability simulations, which required editing the source code). This code is available at: https://github.com/BioGearsEngine/core as of commit #1979440a57b.

The scenario definitions used to generate data, the saved engine states, and the results of these simulations can be found in the in the BioGears Github Repository (https://github.com/BioGearsEngine/paperData/tree/master/SepsisPublicationData).

## Author Contributions

MM designed the BioGears Sepsis Model, generated simulation results, wrote the primary manuscript, and compiled the completed version. JK assisted with drafting the Background and Discussion. He provided invaluable clinical insight with respect to model development, validation, and results interpretation. SW is the lead BioGears software engineer. He is responsible for maintaining and improving BioGears system architecture. He contributed to the writing of the BioGears Architecture and Design section. AB is the Principal Investigator of the BioGears project and has made extensive model contributions to BioGears. He critically reviewed each draft of this manuscript.

### Conflict of Interest

MM, SW, and AB are employed by Applied Research Associates. The remaining author declares that the research was conducted in the absence of any commercial or financial relationships that could be construed as a potential conflict of interest.
